# High-Yield Human Induced Pluripotent Stem Cell-Derived Monocytes and Macrophages Are Functionally Comparable With Primary Cells

**DOI:** 10.3389/fcell.2021.656867

**Published:** 2021-04-13

**Authors:** Di Cui, Alexandra Franz, Sophie A. Fillon, Linda Jannetti, Timo Isambert, Katrin Fundel-Clemens, Heinrich J. Huber, Coralie Viollet, Alexander Ghanem, Akira Niwa, Bernd Weigle, Stefan Pflanz

**Affiliations:** ^1^Boehringer Ingelheim Pharma GmbH & Co. KG, Cancer Immunology and Immune Modulation, Biberach an der Riss, Germany; ^2^Boehringer Ingelheim Pharma GmbH & Co. KG, Medicinal Chemistry, Biberach an der Riss, Germany; ^3^Boehringer Ingelheim Pharma GmbH & Co. KG, Global Computational Biology and Digital Sciences, Biberach an der Riss, Germany; ^4^Center for iPS Cell Research and Application, Kyoto University, Kyoto, Japan; ^5^Boehringer Ingelheim Pharma GmbH & Co. KG, Venture Fund, Ridgefield, CT, United States

**Keywords:** macrophages, monocytes, iPSC, differentiation, myeloid, Crispr/Cas, gene-editing

## Abstract

Macrophages are pivotal effectors of host immunity and regulators of tissue homeostasis. Understanding of human macrophage biology has been hampered by the lack of reliable and scalable models for cellular and genetic studies. Human induced pluripotent stem cell (hiPSC)-derived monocytes and macrophages, as an unlimited source of subject genotype-specific cells, will undoubtedly play an important role in advancing our understanding of macrophage biology and implication in human diseases. In this study, we present a fully optimized differentiation protocol of hiPSC-derived monocytes and granulocyte-macrophage colony-stimulating factor (GM-CSF) or macrophage colony-stimulating factor (M-CSF). We present characterization of iPSC-derived myeloid lineage cells at phenotypic, functional, and transcriptomic levels, in comparison with corresponding subsets of peripheral blood-derived cells. We also highlight the application of hiPSC-derived monocytes and macrophages as a gene-editing platform for functional validation in research and drug screening, and the study also provides a reference for cell therapies.

## Introduction

Monocytes and macrophages, as a central part of the host immune system, play essential roles in homeostasis and inflammation. Monocytes are circulating in the bloodstream and polarize into pro-inflammatory or anti-inflammatory macrophages while migrating to defined tissue locations as directed by various stimuli. Monocytes can be primed with granulocyte-macrophage colony-stimulating factor (GM-CSF) or macrophage colony-stimulating factor (M-CSF) (Lacey et al., 2012; Jaguin et al., 2013). The macrophages derived from GM-CSF- or M-CSF-treated monocytes show different gene expression profiles, and a pro-inflammatory or anti-inflammatory cytokine profile (Rogers and Schwartz, 2018). They were referred to as GM (GM-CSF-exposed macrophages) or MM (M-CSF-exposed macrophages) in this paper. Cumulative studies have shown that the impaired functions of monocytes or macrophages can be associated with metabolic disorders (Aridgides et al., 2019; Barman and Koh, 2020), autoimmune diseases (Rosenblum et al., 2015; Shapouri-Moghaddam et al., 2018), and exogenous infections (Weiss and Schaible, 2015; Galvão-Lima et al., 2017). Thus, the study of human monocytes or macrophages function broadens our understanding of basic immunology but also advances concepts for therapeutic intervention strategies in many human diseases.

The commonly used resource for *in vitro* experiments with human cells is macrophages derived from monocytes isolated from donor blood, which has the advantage of using patient-specific cells regarding the pathophysiology of different diseases. Monocyte counts in human blood are approximately 0.2–0.6 million/ml, representing roughly 10–20% of total peripheral blood mononuclear cells (PBMCs). The limited number of monocytes to be recovered from a blood donation in many settings does not permit the scaling needed for systematic assessment of multiple experimental conditions or compound screening. Besides, the inter-donor variation is also an important factor that can affect the reproducibility of results. Another alternative way preferred by many researches is using the immortalized cells like THP-1 or U937 cell line (Rogers and Schwartz, 2018). Though THP-1 cells are of the same lineage, with the advantages of less variation and unlimited numbers, they exhibit a different morphology and response to stimulations (Bosshart and Heinzelmann, 2016). U937 is a pro-monocytic cell line (Sundström and Nilsson, 1976), displaying a lower phagocytic capacity than human monocyte-derived macrophages (MDMs) or THP-1 macrophages (Mendoza-Coronel and Castanon-Arreola, 2016). Moreover, stemming from human malignant cells raises a high risk of experimental biases that the cells might behave differently from primary cells when interacting with the respective experimental conditions (Schildberger et al., 2013; Chanput et al., 2014). Meanwhile, only the bulk genetic manipulation is accessible on these cells, so the population is not homogenous, and efficiency depends on the genetic manipulation methods or batches. Therefore, a reliable and scalable source of monocytes is highly demanded.

Human embryonic stem cells (HESCs) and human induced pluripotent stem cells (hiPSCs) are well known for the self-renewal and differentiation abilities into all types of cells, including myeloid lineage cells. Therefore, the pluripotent stem cells not only can serve as unlimited clonal source of *in vitro* human myeloid cell models and potentially for cell therapies but also can provide gene-modified cell models. Accumulating studies have shown the potential of hiPSC-derived monocytic like cells in disease-associated research and therapeutic modeling (Karlsson et al., 2008; Senju et al., 2011; Takamatsu et al., 2014; Haenseler et al., 2017; Ackermann et al., 2018; Hong et al., 2018). To date, some publications have already reported the methods of driving pluripotent stem cells into monocyte-like cells and further differentiation into macrophages or dendritic cells (Orlovskaya et al., 2008; Yanagimachi et al., 2013; Cao et al., 2019; Gutbier et al., 2020). However, some methods differentiated the cells by generating embryonic bodies (Gutbier et al., 2020), which require reseeding and performing the size control, and some methods used a feeder-dependent system (Orlovskaya et al., 2008), the batch variation of which highly depends on the serum and feeder cells. Meanwhile, using the feeders or serum from animal sources adds additional biological and regulatory complexities to the path toward possible clinical therapy utility. The other protocols have used serum-free culture conditions (Cao et al., 2019), while the yield of which is rather low, which limits the scale of the studies. In this study, we developed an improved method of differentiating hiPSCs under serum-free condition into monocytes, in a large scale, without the embryonic body differentiation step. The hematopoietic stem cells or monocytes in the middle step can be further expanded and cryopreserved for future use. Therefore, our new protocol provides a reliable, easily scalable, and gene-editable system for human monocyte and macrophage research.

## Results

### Differentiation of Human Induced Pluripotent Stem Cell-Derived CD14-Positive Monocytes

**FIGURE 1:**
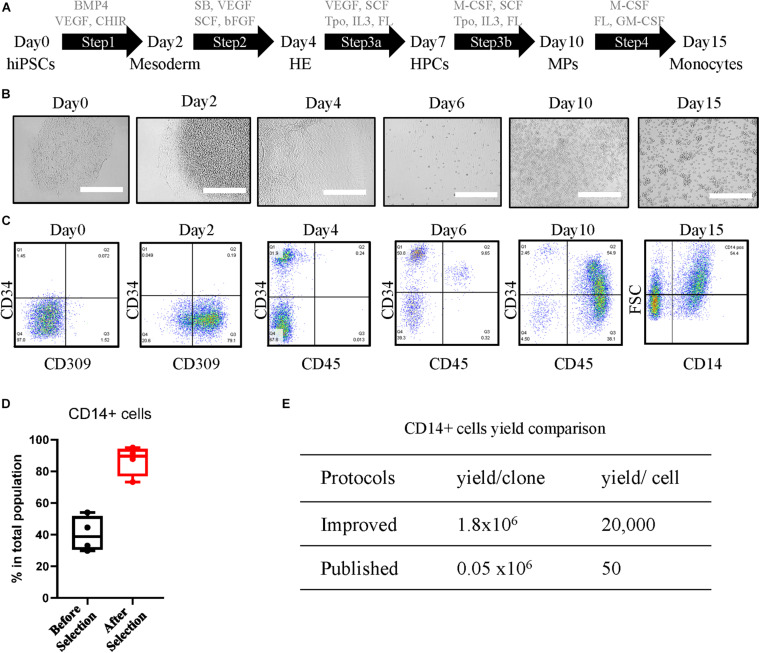
Differentiation and characterization of human induced pluripotent stem cell (hiPSC)-derived CD14-positive monocytes. **(A)** Differentiation schematic overview indicating the cytokines, chemokines, and chemicals for each step: undifferentiated hiPSCs, mesoderm, hemogenic endothelium (HE), hematopoietic progenitor cells (HPCs), myeloid progenitors (MPs), and monocytes. The concentration of the compounds used is shown in *Experimental Procedures* section. **(B)** Representative images of cellular morphology during differentiation of each step at Day 0, Day 2, Day 4, Day 6, Day 10, and Day 15. Scale bar represents 400 μm. **(C)** Characterization of differentiation-specific markers at each stage by fluorescence-activated cell sorting (FACS). **(D)** Yield of CD14-positive cells of the total population before and 2 days after CD14 bead selection. **(E)** Yield comparison of CD14 + cells between improved protocols and other published methods.

The differentiation protocol is fully optimized and largely improved in aspects of the timing of steps and additional cytokines, chemokines, or chemicals, compared with the previously published protocol (Yanagimachi et al., 2013; Matsubara et al., 2019; Ohta et al., 2019; Kase et al., 2020). The differentiation procedure (Figure 1A) was carried out in serum-free conditions. Undifferentiated hiPSCs were maintained in StemFit Basic 2 medium supplemented with basic fibroblast growth factor (bFGF). This step enables the differentiation of single cell-derived hiPSC clones (Figure 1B, Day 0) into mesoderm (Figure 1B, Day 2) with the growth factors of bone morphogenetic protein 4 (BMP4), vascular endothelial growth factor A (VEGFA), and GSK-3 inhibitor CHIR99021 in Essential 8 medium from Day 0 to Day 2. Hemogenic endothelium (HE) (Figure 1B, Day 4) was introduced in combination with growth factors VEGF, TGF-beta/ALK inhibitor SB431542, stem cell factor (SCF), and bFGF for 2 days in Essential 6 medium. Hematopoietic progenitor cells (HPCs) started to emerge as floaters around the organoid (Figure 1B, Day 6) in Stem line II medium supplemented with VEGF, SCF, thyroid peroxidase (Tpo), interleukin (IL)-3, and FLT-3 Ligand (FL) from Day 4 to Day 6. From this stage, the half-medium change was applied. HPCs were further differentiated into myeloid progenitors (MPs) (Figure 1B, Day 10) with additional M-CSF as well as SCF, Tpo, IL-3, and FL in Stem line II medium for 4 days. Robust monocytes (Figure 1B, Day 15) were obtained at Day 15 by 5 day differentiation with M-CSF, GM-CSF, and FL.

The iPSC line 201B7 generated by retroviral transduction from human fibroblasts as previously described (Takahashi et al., 2007) was used in optimizing the protocol. After step 1, around 80% of the cells were positive for endothelial cell-specific marker CD309 (Figure 1C, Day 2). At Day 4 after step 2, more than 30% of the cells expressed the hematopoietic stem cell marker CD34 (Figure 1C, Day 4). The ratio of CD34 was further increased to over 60% in step 3a, with the lymphocyte marker CD45 showing up (Figure 1C, Day 6). The cells at HPC stage can be further expanded in step 3a medium for an additional 7 days from 6 million up to around 18 million per six-well plate with almost 90% CD34 purity (Supplementary Figure 1A), and the cells can be cryopreserved at this stage and further differentiated into CD14 + monocytes (Supplementary Figure 1B). At step 3b, M-CSF was induced to trigger the MP cell differentiation toward classic monocytes. CD45 expression was increased strongly in more than 90% of the cell population (Figure 1C, Day 10). In step 4, GM-CSF was added to promote monocyte proliferation. The classic monocyte population was characterized by CD14 at Day 15 (Figure 1C, Day 15), which counts around half of the total population, and enriched by CD14-coated beads until to more than 90% CD14-positive cells were achieved (Figure 1D). From a single batch, around 18 million CD14-positive cells can be harvested from seeded 900 single cells, starting with 100 clones in one T75 flask. The yield of our protocol was around 1.8 × 10^6^ per single clone or 2 × 10^4^ per seeded hiPSC, which is over 30-fold more than the previous protocol that reported 1.6 × 10^6^ per 30 clones (Yanagimachi et al., 2013) or almost 400-fold of the other protocol with a yield of 50 monocytes per seeded cell (Cao et al., 2019; Figure 1E). The monocytes can be directly used after 2 day recovery for further differentiation and functional assays or cryopreserved as stocks, and the thawed cells expressed typical monocytes markers CD45, CD11b, and CD14 at a level similar to that of fresh iPSC monocytes (Supplementary Figure 1C).

### Human Induced Pluripotent Stem Cell Monocyte-Derived Macrophages Present a Transcriptional Profile Similar to That of Blood Monocyte-Derived Macrophages

**FIGURE 2:**
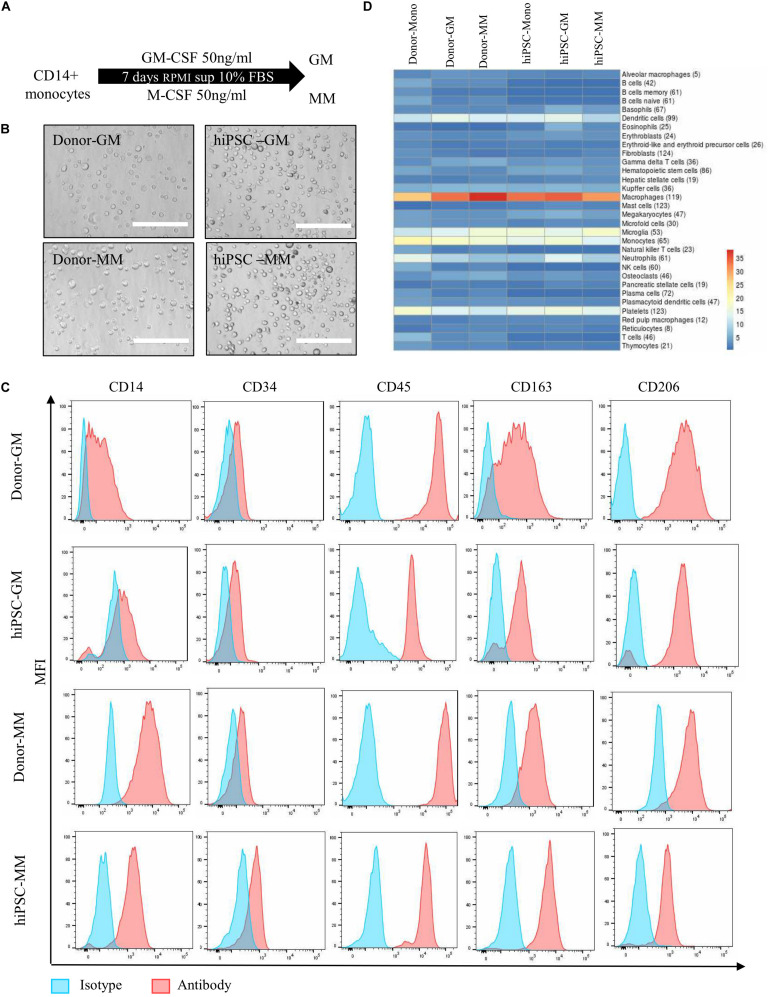
Differentiation and characterization of human induced pluripotent stem cell (hiPSC)-derived monocytes to macrophages. **(A)** Differentiation overview of GM and MM macrophages from hiPSC-derived or donor-derived CD14 + monocytes. **(B)** Representative images of cell morphology from GM and MM macrophages in donor and hiPSC groups. Scale bar represents 200 μm. **(C)** Macrophage marker expression level between hiPSC-derived macrophages and donor-derived macrophages. **(D)** Heatmap presents cell type marker analysis of donor- and iPSC-derived monocyte, and GM and MM macrophages differentiated from relative monocytes. Color scale reflects log10 (*p*-value) of Wilcoxon rank sum test comparing expression levels of cell type marker genes for each cell type against all other cell types. Numbers in parentheses refer to the number or marker genes for the respective cell type.

The typical characterization of monocytes is that they undergo polarization into subtypes of macrophages in response to different stimuli, including pro-inflammatory macrophages or anti-inflammatory macrophages. Our protocol is further extended for differentiation of CD14 + monocytes into macrophages. GM or MM were introduced with GM-CSF or M-CSF, respectively (Figure 2A). To characterize the polarization of the macrophages, the comparison with donor monocyte-derived macrophages was carried out in aspects of cell morphology, typical subtypes surface markers on protein level, and transcriptional profile by next-generation sequencing (NGS) on the RNA level (Table 1). The hiPSC-derived GM and MM have shown similar size and shape as donor macrophages under bright field (Figure 2B).

**TABLE 1:** Overview of the RNA samples for NGS study.

Cell type	Differentiation status	Replicate count
Primary myeloid cell	Monocyte	6
Primary myeloid cell	GM macrophage	6
Primary myeloid cell	MM macrophage	6
iPSC	Monocyte	5
iPSC	GM macrophage	5
iPSC	MM macrophage	5

**NGS, next-generation sequencing; iPSC, induced pluripotent stem cell.**

Both donor and hiPSC GM/MM expressed high levels of CD14 and CD45 as surface markers as monocyte lineage and lower levels of CD34 compared than did HPCs. CD163 and CD206 were all detected as typical macrophage markers, as expected, though with some expression level variations in donor or hiPSC monocyte-derived macrophages (Figure 2C). In order to have a broader view, NGS for characterization of the global transcriptome was carried out on all cell samples with the viability of over 90%. Analyzing the global transcriptome results by principal component analysis (PCA) revealed a clear separation of monocytes, GM and MM in both donor- (Supplementary Figure 2A) and hiPSC-derived cells (Supplementary Figure 2B); the replicates within a sample group fall closely together; i.e., the replicates return very consistent data, indicating the efficient and reproducible polarization process of macrophages. A cell type analysis was carried out with the goal of assessing the transcriptional similarity between the investigated cells and a broad panel of known primary cell types (Figure 2D). Based on computational integration of NGS data for gene signatures, the analysis shows overall high expression levels of macrophage- and monocyte-associated gene signatures, reviewing that the hiPSC-derived cells, just as the donor-derived cells, have a transcriptional profile with a strong macrophage and monocyte component. Furthermore, the cell type analysis is very similar between hiPSC- and donor-derived cells, indicating that they have a comparable macrophage-specific transcriptional profile, particularly GM.

Additionally, we have shown that M0, M1, and M2 (M2a or M2c) macrophages were able to induced from hiPSC monocytes (Supplementary Figure 3A). As expected, hiPSC-M1 has shown a higher expression level of CD80, and hiPSC-M2 macrophages have shown higher expression levels of CD163 and CD206 (Supplementary Figure 3B), similarly as donor-derived macrophages (Supplementary Figure 3C).

### Functional Comparison Between Human Induced Pluripotent Stem Cell- and Donor-Derived Monocytes

**FIGURE 3:**
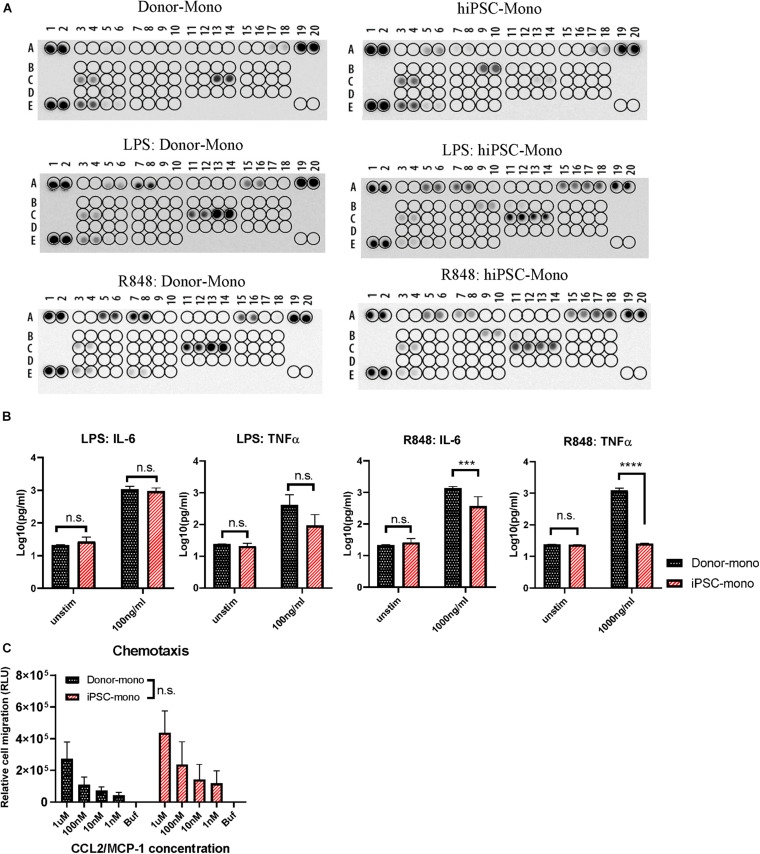
Functional comparison between human induced pluripotent stem cell (hiPSC)- and donor-derived monocytes. **(A)** Cytokine release signature (A5, A6: CCL2/MCP-1; A7, A8: CCL3/CCL4; A15, A16: CXCL1/GROα; A17, A18: CXCL10/IP-10; B9, B10: GM-CSF; C3, C4: IL-1ra/IL-1F3; C11, C12: IL-6; C13, C14: IL-8; E3, E4: MIF/GIF/DER6; E7, E8: TNFα) detected by Proteome Profiler Array with or without stimuli lipopolysaccharide (LPS) (100 ng/ml) or R848 (1,000 ng/ml) for 24 h in hiPSC- and donor-derived monocytes. **(B)** ELISA analysis of IL-6 and TNFα secreted by hiPSC- and donor-derived monocytes under unstimulated condition or upon 24 h stimulation of LPS (100 ng/ml) or R848 (1,000 ng/ml), *n* = 3. **(C)** Chemotaxis responding to CCL2 at gradient concentration of 1 μM, 100, 10, and 1 nM in hiPSC- and donor-derived monocytes, *n* = 3. Two-way ANOVA with Bonferroni correction for multiple comparison: ^∗∗∗^*p* < 0.001, ^∗∗∗∗^*p* < 0.0001.

Monocytes are involved in immune regulation by cytokine and chemokine production upon different stimuli and by migration to the tissue sites under conditions of local tissue distress. Hence, we firstly examined the function of monocytes derived from hiPSC by cytokine secretion signature with or without stimulations of lipopolysaccharide (LPS) and R848, which are ligands of TLR4 and TLR8, representing microbial-like stimuli. The hiPSC monocytes expressed a similar cytokine signature as donor-derived monocytes, containing chemokine (C-C motif) ligand 2/monocyte chemoattractant protein 1 (CCL2/MCP-1), CCL3/CCL4, CXCL1/GROα, CXCL10/IP-10, IL-1ra/IL-1F3, IL-8, MIF/GIF/DER6, and TNFα (Figure 3A, top), while also different in some cytokines like higher GM-CSF and lower IL-8. After a 24-h stimulation of LPS or R848, the cytokine and chemokine secretion showed a pro-inflammatory pattern with higher IL-6, IL-8, CCL2/MCP-1, CCL3/CCL4, and CXCL1/GROα in both hiPSC- and donor-derived monocytes (Figure 3A, bottom), while with CXCL10/IP-10, it is specifically higher in hiPSC-derived monocytes.

ELISA was carried out to further quantify the concentration of IL-6 and TNFα in both groups upon 24 h stimulation of LPS or R848. In response to LPS, IL-6 and TNFα secretion of hiPSC-derived monocytes was comparable without significant difference with that of donor-derived monocytes (Figure 3B, left). hiPSC-derived monocytes also responded to R848 stimuli, while with less IL-6 secretion than the donor group, and almost no TNFα (Figure 3B, right), which is different from donor-derived monocytes.

Another key characteristic of monocytes is that they can migrate along chemokine gradients. CCL2 is also known as MCP-1. CCL2 functions as chemokines recruiting monocytes to the inflammation area by the surface receptors of CCR2 and CCR4. We next evaluated the chemotaxis of hiPSC- and donor-derived monocytes with a CCL2 concentration gradient. hiPSC-derived monocytes exhibited a similar migration ability with increasing CCL2 concentration as primary monocytes (Figure 3C).

### Functional Comparison of Macrophages From Human Induced Pluripotent Stem Cell-Derived and Donor-Derived Monocytes

**FIGURE 4:**
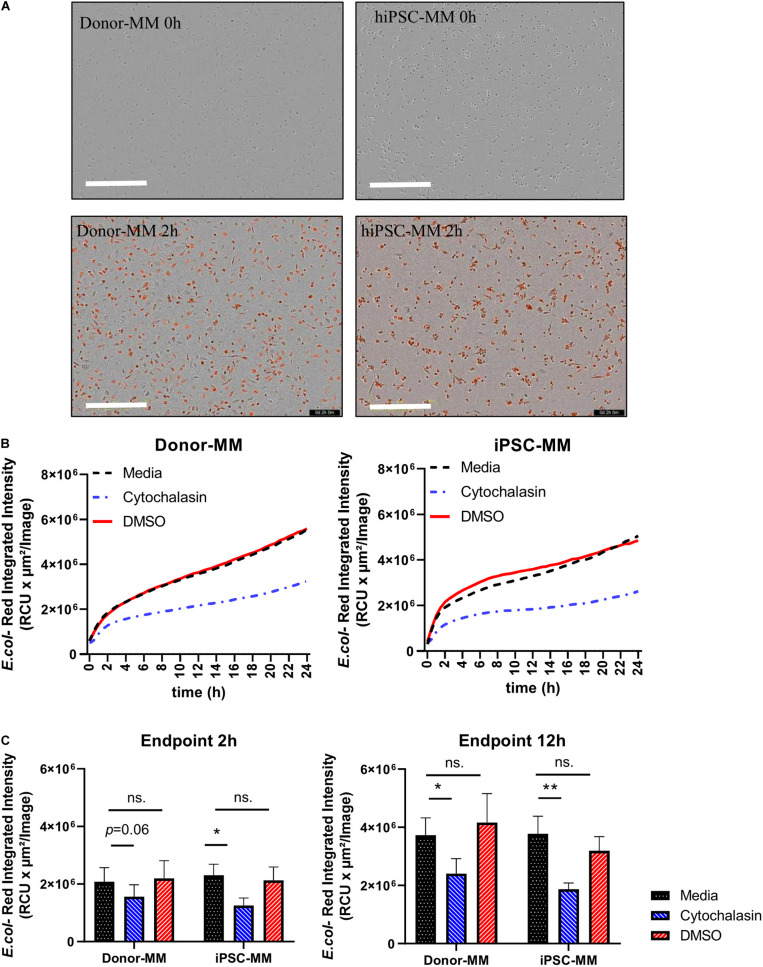
Functional comparison of macrophages differentiated from human induced pluripotent stem cell (hiPSC)-derived and donor-derived monocytes. **(A)** Representative images of *Escherichia coli*-Red uptaking in phagocytosis assay at time points of 0 and 2 h. Scale bar represents 400 μm. **(B)** Real-time graph of *E. coli*-Red integrated intensity from 0 to 24 h under conditions of medium, cytochalasin, and dimethyl sulfoxide (DMSO) groups in hiPSC and donor groups (*n* = 4). **(C)** Analysis of phagocytosis assay at time points of 2 and 12 h in hiPSC and donor groups. Two-way ANOVA with Bonferroni correction for multiple comparison: ^∗^*p* < 0.05, ^∗∗^*p* < 0.01.

Phagocytosis of microbes or cell debris is perhaps the paramount function of macrophages. Hence, we examined the phagocytosis activity of macrophages derived from hiPSC monocytes (hiPSC-MDM). MM were obtained by 7 day differentiation with M-CSF. Cytochalasin D, as an inhibitor known to block phagocytosis, was added to macrophages as a negative control as well as dimethyl sulfoxide (DMSO). Afterward, macrophages were exposed to pHrodo-labeled *Escherichia coli* BioParticles (4669, Sartorius) for 24 h. A clear red signal was observed at 2 h in both hiPSC and donor MDM groups (Figure 4A). The real-time graph of *E. coli*-Red integrated intensity was highly identical between hiPSC and donor MDM groups, as time increased in the media and DMSO groups, while they were largely inhibited by cytochalasin (Figure 4B). The phagocytosis activity showed no statistically significant differences between media and DMSO groups at the 2-h time point, while it tended to be lower with cytochalasin, which was similar in hiPSC or donor MDMs (Figure 4C, left). The value of Red integrated intensity was doubled at the time point of 12 h (Figure 4C, right) compared with that of 2 h, and a more significant inhibition by cytochalasin was observed, without being affected by DMSO as controls. HiPSC-MDMs showed comparable phagocytosis ability regarding *E. coli* particles as donor MDMs.

### Crispr/Cas Knockout and of Immune Regulatory Receptors in Human Induced Pluripotent Stem Cells

**FIGURE 5:**
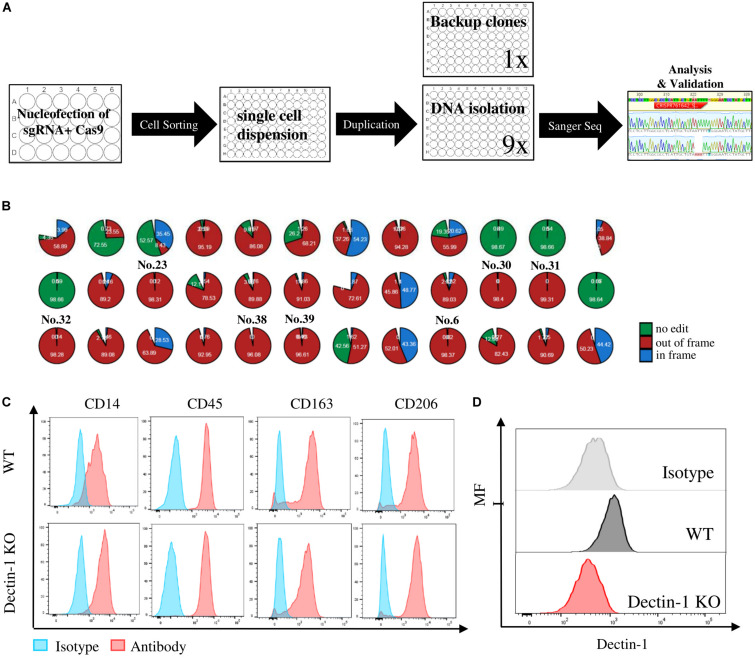
Crispr/Cas knockout (KO) and of immune regulatory receptors in human induced pluripotent stem cells (hiPSCs) and functional analysis of macrophages derived from relative KO clones. **(A)** Workstream scheme for generating and analyzing of single cell KO clones in hiPSC. **(B)** Eight out-of-frame homozygous Dectin-1 KO clones were identified by Sanger sequencing and interference of CRISPR edits (ICE) analysis. **(C)** Macrophage marker expression level between wild type (WT) and Dectin-KO clone No. 32-derived macrophages. **(D)** Dectin-1 expression level and relative isotype in macrophages derived from WT and Dectin-1 KO clone No. 32.

Myeloid cells are evaluated for their potential as therapeutic target cells in various diseases; hence, it is important to achieve efficient genetic manipulation of genes of interests on these cells for functional study or phenotypic screening. However, gene-editing efficiency with the current approaches is particularly difficult in myeloid-lineage cells, likely due to sensitivity to the foreign materials (Hornung et al., 2006; Leyva et al., 2011; Bobadilla et al., 2013). On the one hand, gene manipulation requires large numbers of primary cells, which limits the scales of the study, and on the other hand, the gene-editing efficiency varies from batch to batch, resulting in a large variation. Therefore, to establish the knockout (KO) iPSC clones of targeted genes, for instance, immune regulatory receptors, it is a better alternative to obtain a homogenous and scalable iPSC-derived myeloid population. Hereby, we established a gene-editing protocol to generate targeted gene KO iPSC clones, as indicated by the workstream (Figure 5A). The KO validation in looking for the out-of-frame homozygous clones was carried out by Sanger sequencing and interference of Crispr edits (ICE). For example, eight out-of-frame homozygous Dectin-1 KO clones were identified from 41 single clones in a single batch, with an efficiency of 19.5% (Figure 5B). The KO region of the eight clones was all at Crispr/Cas genome editing region, as expected (Supplementary Figure 4). The KO clone, e.g., No. 32, was successfully differentiated into macrophages derived from monocytes as our established protocol, as well as the wild-type clone (Figure 5C), indicating that Dectin-1 is not required for the differentiation process. Fluorescence-activated cell sorting (FACS) analysis using an anti-Dectin-1 antibody confirmed the successful KO of Dectin-1 in macrophages (Figure 5D).

## Discussion

Monocytes or macrophages are increasingly explored as possible cellular targets for pharmacological intervention in various diseases. Recently, macrophages engineered with chimeric antigen receptors (CARs) have been described as therapeutic cells in solid tumors (Klichinsky et al., 2020). Currently, the main source of monocytes or macrophages is primary cells from blood, the variations of which largely depends on the donor individual conditions. Moreover, the numbers of primary cells usually do not allow large-scale studies or screens, and even much less for cell therapies. Though many studies use immortalized cells, like THP-1, to study the function of macrophages, they entail responses upon stimuli differently from the primary cells (Bosshart and Heinzelmann, 2016), and results obtained with THP-1 frequently require subsequent verification in a more translational setting with primary cells.

To overcome the issue of donor variability, and to obtain a reproducible and scalable source for human myeloid cells, we developed and described an improved method in generating hiPSC-derived monocytes. Different from recently reported protocols of monocyte differentiation (Gutbier et al., 2020), we used the 2D culture setting, starting from the single clones instead of the 3D culture of embryonic bodies. The hiPSCs were firstly induced into mesoderm and further differentiated into HE and HPCs with high efficiency (Matsubara et al., 2019; Ohta et al., 2019; Kase et al., 2020), and at this stage, the cells can be expanded for an additional 7 days and conveniently cryopreserved depending on the scaling and timing demands of the following experimental procedures and scientific objectives. The HPCs were then further directed to MPs and monocytes in 10 days. The monocytes were characterized with CD45, CD11b, and CD14 markers. The integrins are important cell–cell interaction regulators for monocytes, the markers of which such as CD49d (ITGA4), CD29 (ITGB1), and CD18 (ITGB2) were also presented in the transcriptional level in NGS results. CD29 and CD18 have shown a similar expression level as donor monocytes, while CD49d exhibited a lower expression, indicating that the maturation of hiPSC-derived monocytes might be different from that of blood circulating monocytes. The CD14 + monocytes can be enriched by CD14 + -coated magnetic beads to over 90% purity. In our protocol, the yield of monocytes production is around 18 × 10^6^ per 900 seeded iPSC single cells or 2 × 10^4^ per seeded hiPSC in one T75 flask. The higher yield allows a larger scale of research studies or compound screening and potentially for cell therapy. A higher yield is believed to be achieved with continuous harvest of HPC floaters; a secondary harvest of the HPC floaters in 3 days still maintains the high purity of over 70%, 12 million per six-well plate of CD34 cells.

The monocytes can be further differentiated into polarized GM or MM. GM-CSF-induced GM were more heterogeneous in both groups, while MM were mostly homogenous. The differentiation medium was not serum-free, which could represent an opportunity for further optimizations using serum replacement. The typical macrophage markers were highly expressed in hiPSC-MDMs, while the monocytes and MP markers were largely reduced as primary cells. These cells show similar gene expression profiles in all genes or unique genes as the primary monocytes and GM or MM. Macrophage markers showed higher expression in primary MDMs than in the iPSC group, likely due to a weaker separation between macrophages and monocytes. More importantly, the cells behaved similar to that of their primary counterparts in different functional activities, including cytokine release, chemotaxis, and phagocytosis. The cytokine secretion signature was almost identical in hiPSC-derived monocytes with the secretion level of a couple of cytokines different from primary monocytes, like higher GM-CSF and lower IL-8, indicating the different basal levels of cytokine or chemokines in the hiPSC and primary groups. LPS and R848 stimulations were used to further evaluate the function of monocytes. The pro-inflammatory cytokine signature was similar in response to LPS as shown in the cytokine array or IL-6 and TNFα quantification, while some cytokines were less in hiPSC monocytes than primary monocytes in response to R848 like TNFα, which indicate that the hiPSC-derived monocytes might be more tolerant toward some stimuli than primary monocytes. Another pivotal function of chemotaxis was also very similar between hiPSC and primary monocytes, that the monocytes were recruited by CCL2/MCP-1 similarly in different concentration conditions. The hiPSC can actively migrate to the inflammation area as primary monocytes, which provides a positive feedback. Adding to the previous study (Yanagimachi et al., 2013), the phagocytic activity of macrophages derived from hiPSC monocytes was tested in comparison with primary MDMs with similar phagocytosis pattern, in line with some other studies using distinct protocols (Cao et al., 2019). Additionally, we have proved that with this improved protocol, hiPSC-derived monocytes can be further introduced into M0, M1, M2a, or M2c, which present the same characterization as donor-derived cells. The purified monocyte can be cryopreserved with a recovery rate of around 70%, higher than that of the another study published (Gutbier et al., 2020), and macrophages derived from the cryopreserved monocytes can phagocytose similarly as primary cells. The cryopreserved monocytes exhibited a similar expression level of CD45, CD14, and CD11b after thawing as fresh hiPSC monocytes, but it had a reduced level of CD16. The dimmer expression of CD16 was also observed in cryopreserved PBMC-derived monocytes compared with fresh isolated monocytes. On the other hand, the cryopreserved monocytes did not perform well in a chemotaxis assay, indicating that the motility was affected by cryopreservation (Abda et al., 1998), but no evidence was shown that the CCR expression was affected by the freezing procedure (Campbell et al., 2001). Different freezing and thawing methods should be tested in the future to optimize the recovery conditions.

Besides the scalable number of cells, another large advantage of using hiPSC-derived macrophages is the feasibility of gene editing. Gene-editing protocols elicit extra stress to the primary cells, especially within the period of time when monocytes differentiate to macrophages in 5–7 days. Moreover, only bulk KO studies can be carried out with primary cells, which may not achieve the functional KO with acceptable homogeneity in many cases. Gene manipulation on hiPSC allowed us to obtain complete KO clones. In this study, we have established a gene KO platform, where single cell sorting was used to guarantee the purity of single clones. Sanger sequencing and ICE were used to quickly define the out-of-frame KO clones. The KO clones were then differentiated into macrophages with a confirmed absence of the target protein. The usage of the technique on hiPSC provides us a scalable and reproducible resource of myeloid functional study on a single gene KO resolution.

In summary, we have developed an improved protocol to differentiate hiPSC into CD14-positive monocytes and monocyte-derived macrophages in a significantly larger scale than previously reported. The cryopreserved monocytes can be used for some functional assays. The hiPSC-derived cells exhibited highly similar transcriptional profiles and performed similarly in functional assays as compared with blood-derived monocytes and monocyte-derived macrophages. Gene editing by Crispr/Cas KO further extends the application of the downstream research in myeloid field. iPSCs represent a versatile platform for production of functional human monocytes and macrophages, which can serve as an attractive alternative to human blood-derived myeloid cells to address biological and pharmacological questions with human cells *in vitro*.

## Experimental Procedures

### Cell Culture

This study used the hiPSC line 201B7 (Takahashi et al., 2007), daughter cell lines including 201B7-Cas9 (Cas9 was inserted by homologues recombination into the AAVS1 locus) (Supplementary Figure 5), and 201B7-Dectin KO cells. hiPSC was cultured as previously described with minor difference for adaption (Yanagimachi et al., 2013). Specifically, around 5,000 single cells per well were seeded and maintained at 37°C with 5% CO_2_ in StemFit Basic02 (ASB01, Ajinomoto) medium supplemented with 10 μg/ml of Y-27632 (10-2301, FOCUS) for the first 48 h and 100 ng/ml of FGF2 (33-FB-025, R&D) in a six-well plate (140675, Thermo Fisher) precoated with laminin iMatrix-511 (892012, Amsbio) at 37°C for 1 h. The medium was changed every other day for a week before being passaged at around 70–80% confluence. The cells were washed once with phosphate-buffered saline (PBS) (70011069) and detached with Trypsin Select (12563, Gibco) for 4 min at 37°C. The Trypsin was neutralized with medium and removed by centrifugation at 300 *g* for 5 min. The cells were then seeded for maintenance or differentiation.

### Differentiation of Monocytes From Human Induced Pluripotent Stem Cells

Two hundred undifferentiated hiPSCs was seeded per well in a laminin-coated six-well plate to generate around 20 colonies in a week. The colony size was measured at around 400–700 nm before the start of differentiation. The larger-scaled differentiation can be carried out in T75 flasks seeded with 900 hiPSCs. At Day 0, the maintenance medium was switched to Essential 8 medium (A1517001, Gibco) supplemented with 80 ng/ml of VEGFA (293-VE-050), 80 ng/ml of BMP4 (314-BP-010), and 4 μM of CHIR99021 (4423/10, TOCRIS). At Day 2, the medium was replaced with Essential 6 medium (A1516401) supplemented with 80 ng/ml of VEGFA, 50 ng/ml of FGF2, 50 ng/ml of SCF (255-SC-050, R&D), and 2 μM of SB431542 (1614/10, TOCRIS). At Day 4, the cells were served with Step4 basic medium, which was Stemline II (S0192, Sigma) with ITSX (51500-056, Gibco) or StemPro-34 medium (10640-019), supplemented with 40 ng/ml of VEGFA, 50 ng/ml of SCF, 10 ng/ml of TPO (288-TPN-025, R&D), 50 ng/ml of IL-3 (203IL-050, R&D), and 50 ng/ml of FLT-3 (308-FK-025/CF, R&D). At Day 7, a half-medium change was carried out with Step4 basic medium supplemented with 50 ng/ml of SCF, 10 ng/ml of TPO, 50 ng/ml of IL-3, 50 ng/ml of FLT-3, and 50 ng/ml of M-CSF (316-MC-100, R&D). At Day 10, Step4 basic medium supplemented with 50 ng/ml of FLT-3, 50 ng/ml of M-CSF, and 25 ng/ml of GM-CSF (215-GM-050, R&D).

### Isolation of Peripheral Blood Mononuclear Cell From Buffy Coat

Buffy coat from six donors (6xHealthy, 4xRh positive, 2xRh negative, 1xO blood type, 5xA blood type) obtained from Deutsches Rotes Kreuz Kreisverband, Poslfach 15 64, 89005 Ulm/Donau, were used for this study. The buffy coat was diluted with two times amount of washing buffer, PBS (10010-015, Gibco) with 2 mM of EDTA (15575-038, Gibco). The diluted buffy coat was centrifuged at 800 *g*, 15 min, room temperature (RT) without brake with Leucosep Tube (227290, Greiner) containing 15 ml of Ficoll Paque pre-spinned below the filter. The interphase was collected and washed once with washing buffer at 300 *g*, 10 min, and twice at 200 *g*, 10 min. Cell pellets were lysed with 10 ml of ACK lysis (A10492-01, Gibco) for 7 min at RT and washed twice with washing buffer at 300 *g*, 5 min. Cell number and viability were counted with Countess II (AMQAX1000, Thermo Fisher) with Trypan blue stain 0.4% (T10282, Thermo Fisher Scientific).

### CD14-Positive Selection

At the end of differentiation of Day 15, the cells were collected and filtered through 30-μm filters. The CD14 + monocytes derived from hiPSC or PBMC were enriched with CD14 antibody-coated magnetic MicroBeads (120-050-201, Miltenyi). The CD14 + cells were selected and cultured at the density of 1 mio/ml, 3 ml per well in six-well up-cell plates (174901, Thermo Fisher) before further functional assay or differentiation.

### Differentiation of Macrophages From Monocytes

The CD14 + -selected monocytes were further differentiated with Roswell Park Memorial Institute (RPMI) 1640 medium (61870010, Gibco) supplemented with 10% fetal bovine serum (FBS) (10500-064, Gibco) and 50 ng/ml of GM-CSF or 50 ng/ml of M-CSF toward GM-CSF- or M-CSF-induced macrophages in 7 days. The cells were ready for functional assays.

M0 macrophages were induced by M-CSF at the concentration of 100 ng/ml for 7 days; M1, M2a, or M2c macrophages were obtained by preincubation with 100 ng/ml of M-CSF for 5 days and then stimulated with 20 ng/ml of LPS and 50 ng/ml of IFNg, 20 ng/ml of IL-4 and 20 ng/ml of IL-13, or 20 ng/ml of IL-10 for an additional 2 days, respectively.

### RNA Isolation and Quality Control

One million cells with viability of over 90% by Trypan blue measurement were collected, centrifuged, washed in PBS, and lysed in RLT buffer (79216, Qiagen) before storage at -80°. RNA was later extracted using the Qiagen RNeasy Mini Kit (74104, Qiagen, United States) according to manufacturer’s specifications. An on-column DNA digestion (DNASE10-1SET, Sigma) was performed; and the final elution volume was 30 μl. Total RNA was quantitatively and qualitatively assessed using the fluorescence-based Broad Range Quant-iT RNA Assay Kit (Q33140, Thermo Fisher) and the Standard Sensitivity RNA Analysis DNF-471 Kit (DNF-471-0500, Agilent) on a 48-channel Fragment Analyzer (Agilent), respectively. Concentrations averaged at 25 ng/μl, while RNA integrity number (RIN) ranged from 8.2 to 10, with a median of 9.9.

### Whole-Transcriptome Profiling With PolyA Enrichment (mRNA-Seq)

Thirty-three human macrophage- and monocyte-derived RNA samples were normalized on the MicroLab STAR automated liquid platform (Hamilton). Total RNA input of 200 ng was used for library construction with the NEBNext Ultra II Directional RNA Library Prep Kit for Illumina #E7760, together with the NEBNext Poly(A) mRNA Magnetic Isolation Module #E7490 upstream and the NEXNext Multiplex Oligos for Illumina #E7600 downstream (all New England Biolabs). The only deviation from the manufacturer’s protocol was the use of Ampure XP beads (Beckman Coulter) for double-stranded cDNA purification, instead of the recommended SPRIselect Beads. mRNA sequencing libraries were quantified by the High Sensitivity dsDNA Quanti-iT Assay Kit (Thermo Fisher) on a Synergy HTX (BioTek). Library molarity averaged at 16 nM. Final library size distribution was also assessed (smear analysis of ∼410 bp average and adapter dimer presence <0.5%) by the High Sensitivity Small Fragment DNF-477 Kit on a 48-channel Fragment Analyzer (Agilent). Out of 33 sequencing libraries, 32 passed quality check and were then normalized on the MicroLab STAR (Hamilton) and pooled and spiked in with PhiX Control v3 (Illumina). The library pool was subsequently clustered with the HiSeq 3000/4000 SR Cluster Kit on a cBot and sequenced on a HiSeq 4000 Sequencing System (Illumina) with dual index, single read at 85 bp length (read parameters: Rd1, 86; Rd2, 8; and Rd3, 8), reaching an average depth of 24 million pass-filter reads per sample (14.6% CV).

### RNA-Seq Data Analysis

Demultiplexing was performed using bcl2fastq v2.20.0.422 from Illumina^1^. Sequencing reads from the RNA-seq experiment were processed with a pipeline building upon the implementation of the ENCODE “Long RNA-seq” pipeline, also described in Schlager et al. (2020): filtered reads were mapped against the *Homo sapiens* (human) genome hg38/GRCh38 (primary assembly, excluding alternate contigs) using the STAR (v2.5.2b) aligner (Dobin et al., 2013), allowing for soft clipping of adapter sequences. For quantification, we used transcript annotation files from Ensembl version 86, which corresponds to GENCODE 25. Gene expression levels were quantified with the above annotations, using RSEM (v1.3.0) (Li and Dewey, 2011) and featureCounts (v1.5.1) (Liao et al., 2014). Quality controls were implemented using FastQC (v0.11.5) (Andrews, 2015), available online at: http://www.bioinformatics.babraham.ac.uk/projects/fastqc/, picardmetrics (v0.2.4) [(Slowikowski, 2016); available online at https://github.com/slowkow/picardmetrics], and dupRadar (v1.0.0) (Sayols et al., 2016) at the respective steps. The PCA was run on vst-transformed counts of the top 500 variance genes.

### Cell Type Marker Analysis

PanglaoDB (Franzén et al., 2019) was downloaded on January 8, 2020. All genes assigned with species “Hs” (for *Homo sapiens*) and flag “canonical marker” set to 1 were used for further analysis. Group median expression levels (tpm) have been determined for each gene and sample group. We applied a Wilcoxon rank sum test comparing median expression levels of marker genes for a given cell type against all others to evaluate enrichment of marker genes in highly expressed genes. For visualization purposes, we applied a *p*-value cutoff for the cell types to be displayed in the heatmap of *p* < 0.01 for all markers.

### Flow Cytometry

The cells were collected and resuspended in blocking buffer, 10% FBS, 2 mM of EDTA, and 2% Fc-block in PBS. The cell density were around 100,000 cells per well in V-bottom 96-well plates (M9686-100EA, Greiner). According to the manufacturers, a certain amount of each antibody and relative isotype controls, including CD14 (301830, BioLegend) and Isotype (400260, BioLegend), CD45 (555485, BD) and Isotype (555751, BD), CD309 (89106, BD) and Isotype (554680, BD), CD34 (343606, BioLegend) and Isotype (558595, BD), CD163 (333632, BioLegend) and Isotype (563330, BD), CD206 (740797, BD) and Isotype (563044, BD), and Dectin-1 (12-9856-42, eBioscience) and Isotype (558595, BD), were added to the cell suspension. Staining was performed in the dark at RT for 10 min. Cells were centrifuged at 300 *g* for 5 min; after removal of the supernatant, the cells were washed with 100 μl of FACS buffer, 10% FBS, and 2 mM of EDTA in PBS and centrifuged again at 300 *g* for 5 min. To each well, 100 μl of FACS buffer was added. The cells can be stored at 4°C or on ice before FACS by LSRFortessa X-20 (BD). The expression level was analyzed by FlowJo V10.5.3.

### Human Cytokine Array Analysis

Supernatants from three independent experiments were collected, and 100 μl of each was mixed pre-experiment; 300 μl of supernatants was used for each cytokine array membrane using Human XL Cytokine Array kit (ARY022B, R&D).

### Enzyme-Linked Immunosorbent Assay

Supernatants from the source of three donors or three independent batches of hiPSC-derived cells were collected, and 50 μl per well in duplicates was used for ELISA. Human IL-6 and TNFα Duoset (DY206, DY210, R&D) were carried out according to the manufacturer’s instructions. The final concentration of the cytokines was calculated with the standard curve.

### Chemotaxis Assay

Set monocyte cell concentration to 2.5 × 10^6^ cell/ml with the buffer of Hank’s Balanced Salt Solution (HBSS) with 0.1% bovine serum albumin (BSA). CCL2/MCP-1 was diluted in the buffer to gradient concentrations of 1 μM, 100 nM, 10 nM, and 1 nM and 0 nM. The CTX plate (101-5, Neuroprobe) was filled 30 μl per well with diluted CCL2/MCP-1 and then sealed with the membrane; 25 μl of the cell suspension was pipetted onto the membrane. The cells were migrated for 2 h in the incubator. After the migration, 15 μl per well cell suspension was transferred from the plate into the half area white plate (6005290, PerkinElmer) with 15 μl of CellTiter-Glo reagent (G7571, Promega) per well, and the plate was incubated for 10 min before being measured with 2104 EnVision multilabel plate reader (PerkinElmer).

### Phagocytosis Assay

Twenty-five thousand macrophages were seeded per well in 96-well plates (655090, Greiner) with 100 μl of medium for 2 h. The cells were treated with medium, 200 nM of cytochalasin or 0.08% DMSO as control for 1 h. pHrodo Red *Escherichia coli* BioParticles (4615, Essen BioScience) were dissolved and sonicated before use and added to macrophages to the final concentration of 100 μg/ml. The final volume in each well was 200 μl. Plates were incubated in an IncuCyte S3, and images were acquired using 10 × objective every 30 min for 24 h. Phagocytosis capacity was analyzed using the IncuCyte module.

### Crispr/Cas Knockout Single-Colony Generation

The Dectin sgRNA were predesigned TrueGuide Synthetic gRNA (Invitrogen) CRISPR761642_SGM, with target DNA sequence of CGCCTCATTGCTGTAATTTT (forward strand). Neon Transfection was carried out by using TrueCut Cas9 protein (A36498, Invitrogen) with Neon Transfection system (MPK5000, Thermo Fisher) and Neon 100 μl kit (MPK10096, Thermo Fisher) according to the instruction at 1,400 V, 10 s, three times. The transfected cells were seeded in a single cell by cell sorting into 96-well plates precoated with laminin. When cells reached 80% confluent, uneven passage with the ratio 9:1 was applied. Ninety percent of the cells were lysed with DirectPCR Lysis Buffer (301-C, Viagen), and PCR analyses were performed without further purification using AccuStart II PCR ToughMix (95142-800, Quantabio) or Phusion High Fidelity DNA Polymerase (F-530, Thermo Fisher). PCRs were purified using MagMax/KingFisher with AMP Pure XP Beads (A63881, Beckman Coulter) and sent to Sanger sequencing (Eurofins). The results were analyzed with ICE. The homogenous KO clones were expanded and frozen as stocks.

### Cell Cryopreservation

Four to five million HPCs or monocytes were frozen in FBS with 10% DMSO in cryopreservation boxes, stored at −80°C overnight, and transferred to −150°C the next day.

### Statistical Analysis

All data values are expressed as means with standard error of the mean (SEM). Experiments were performed at least three times. Statistical significance of ELISA and chemotaxis was determined by two-way ANOVA with Bonferroni correction for multiple comparison: ^∗∗∗^*p* < 0.001; ^∗∗^*p* < 0.005; and ^∗^*p* < 0.05 (compared with LPS stimulation).

## Data Availability Statement

RNA-seq data supporting the results in this article can be found at https://www.ncbi.nlm.nih.gov/geo/query/acc.cgi?acc=GSE165152.

## Ethics Statement

All studies on human donor blood were performed in accordance with the guidelines and regulations of German legislation, and the experimental protocol was approved by the Ethical Committee of the Landesaurztekammer Baden-Wuerttemberg (Germany). Anonymized blood samples were obtained from healthy volunteers who provided written informed consent.

## Author Contributions

DC, SP, and BW: conceptualization. DC, AF, SF, LJ, TI, AG, and AN: methodology. DC, BW, KF-C, HH, CV, and AG: data analysis. DC: writing of the manuscript draft. DC, SF, KF-C, CV, AG, and SP: writing and editing. SP and BW: project supervision. All authors have read and agreed to the published version of the manuscript.

## Conflict of Interest

DC, AF, SF, LJ, TI, KF-C, HH, CV, AG, BW, and SP were employed by the company Boehringer Ingelheim Pharma GmbH. The remaining author declares that the research was conducted in the absence of any commercial or financial relationships that could be construed as a potential conflict of interest.

## Footnotes

1 https://emea.support.illumina.com/downloads/bcl2fastq-conversion-software-v2-20.html
