# Supplementary figures and images for: High-Yield Human Induced Pluripotent Stem Cell-Derived Monocytes and Macrophages Are Functionally Comparable With Primary Cells
Source: Front Cell Dev Biol. 2021 Apr 13;9:656867. doi: 10.3389/fcell.2021.656867 (PMC8080307; doi:10.3389/fcell.2021.656867)

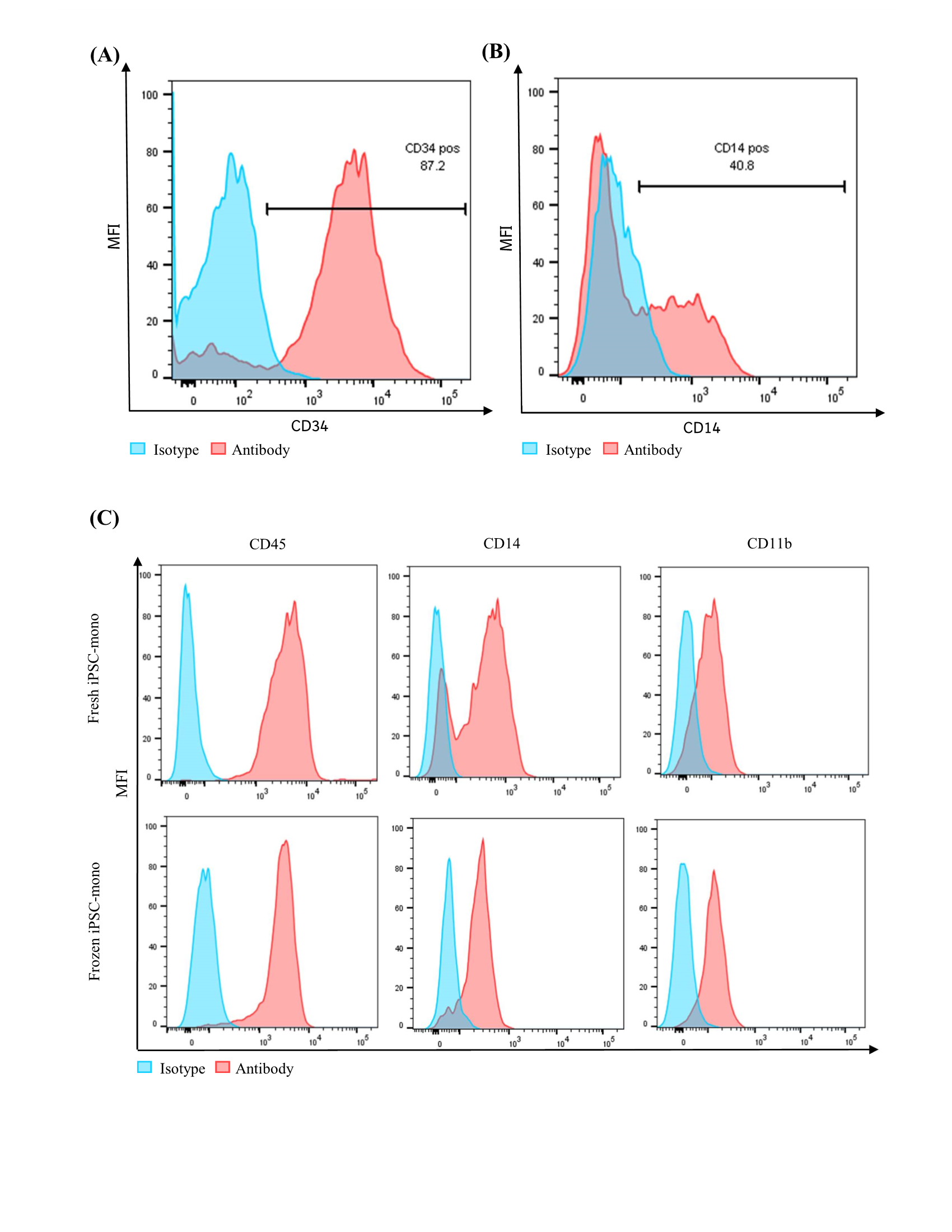

Supplement: Supplementary file 1 [file Image_1.TIFF]

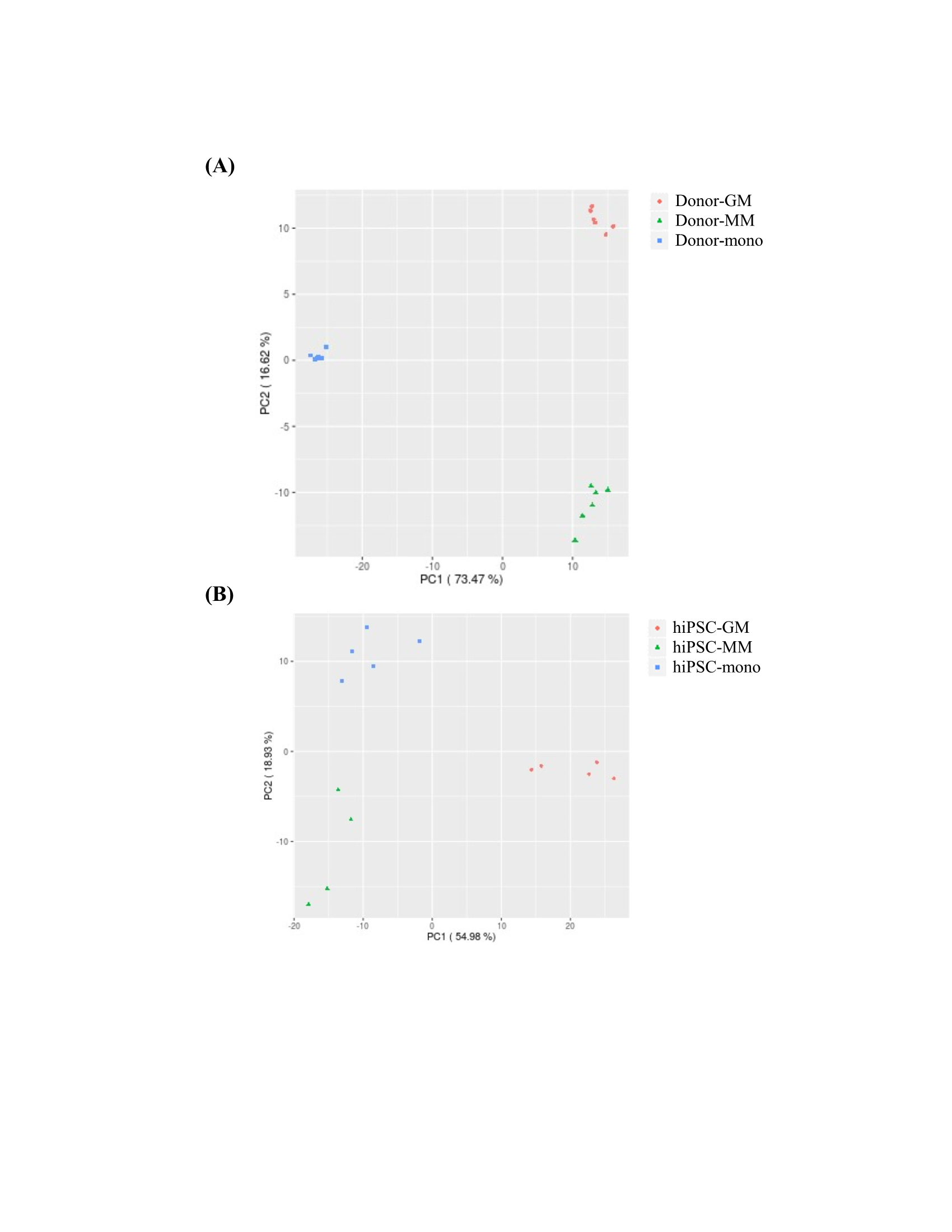

Supplement: Supplementary file 2 [file Image_2.TIFF]

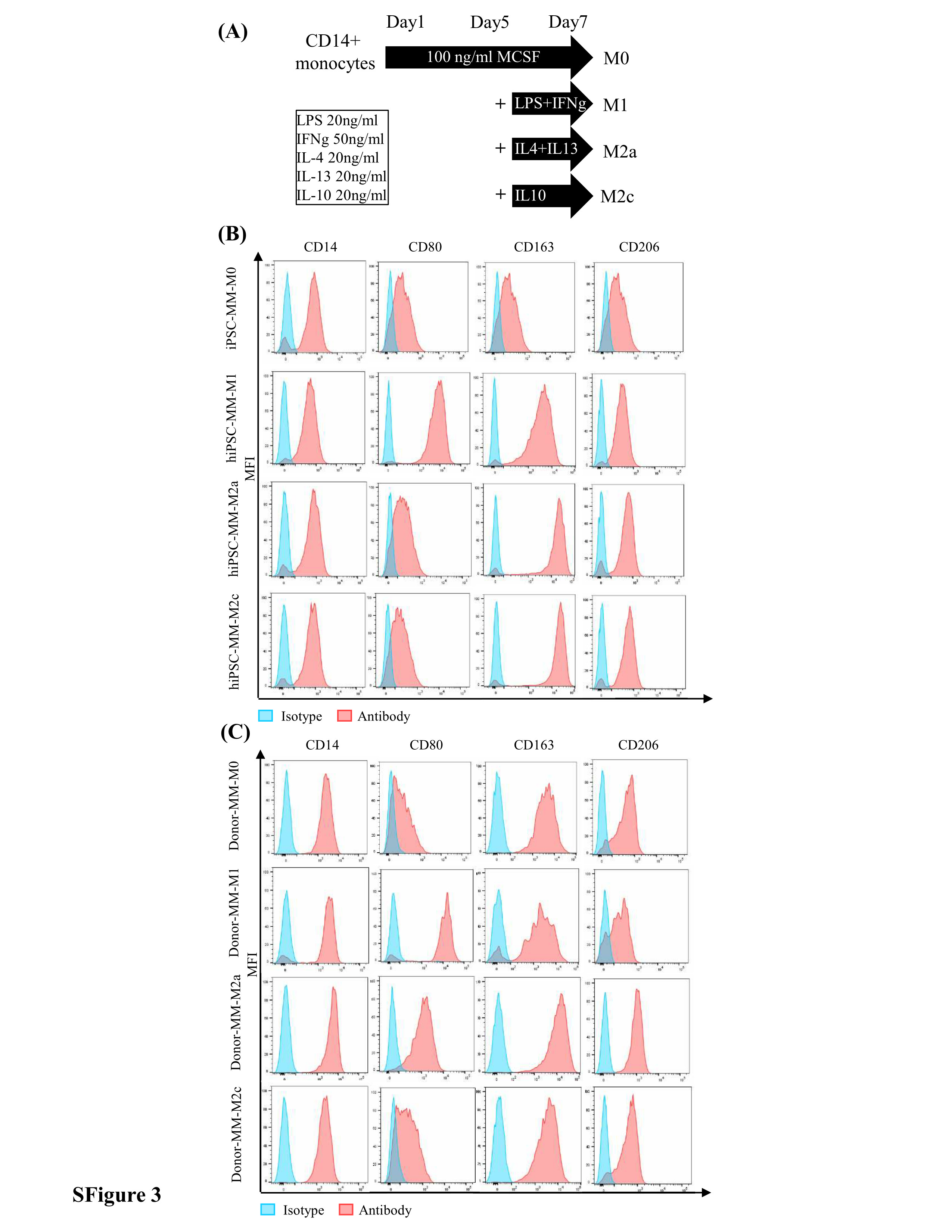

Supplement: Supplementary file 3 [file Image_3.tif]

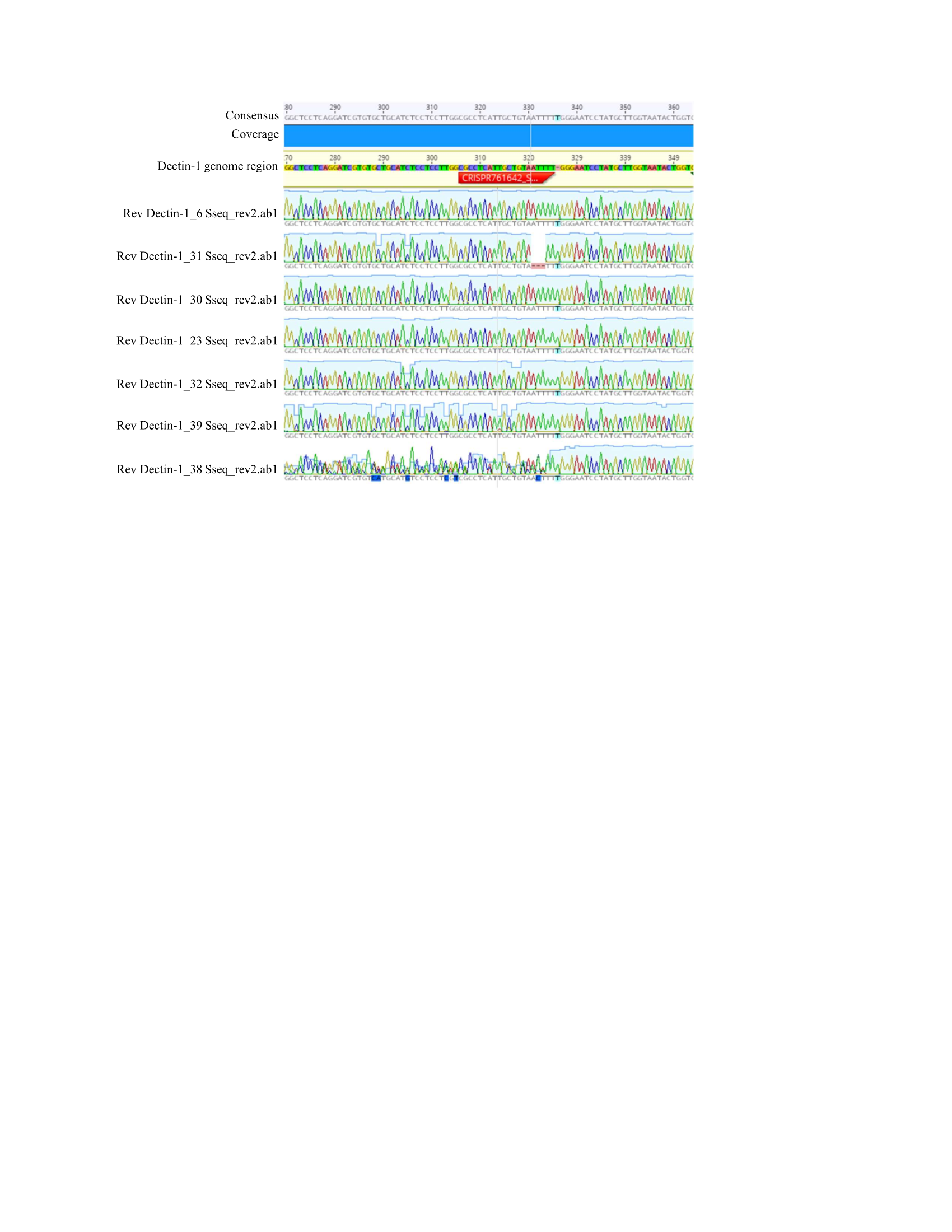

Supplement: Supplementary file 4 [file Image_4.TIFF]

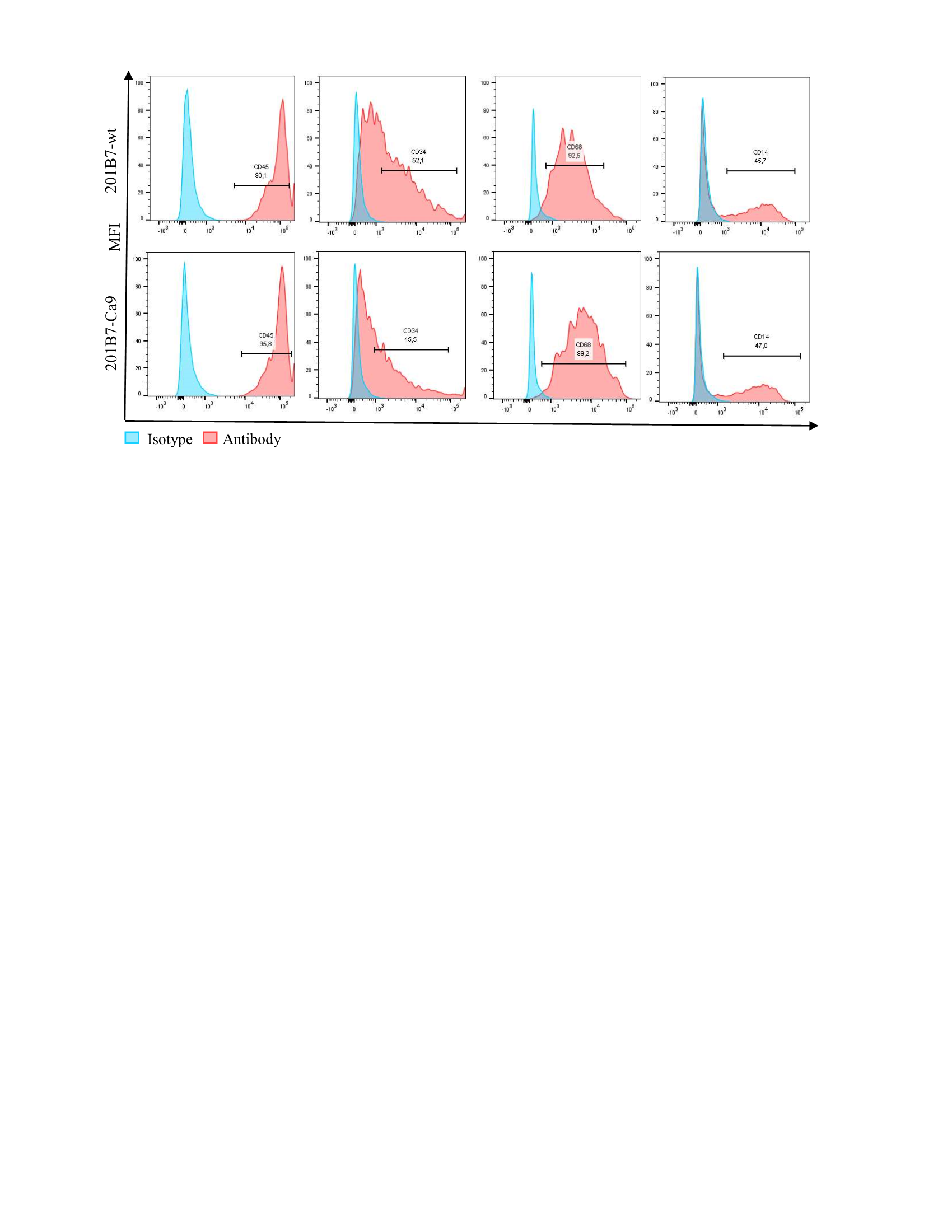

Supplement: Supplementary file 5 [file Image_5.TIFF]

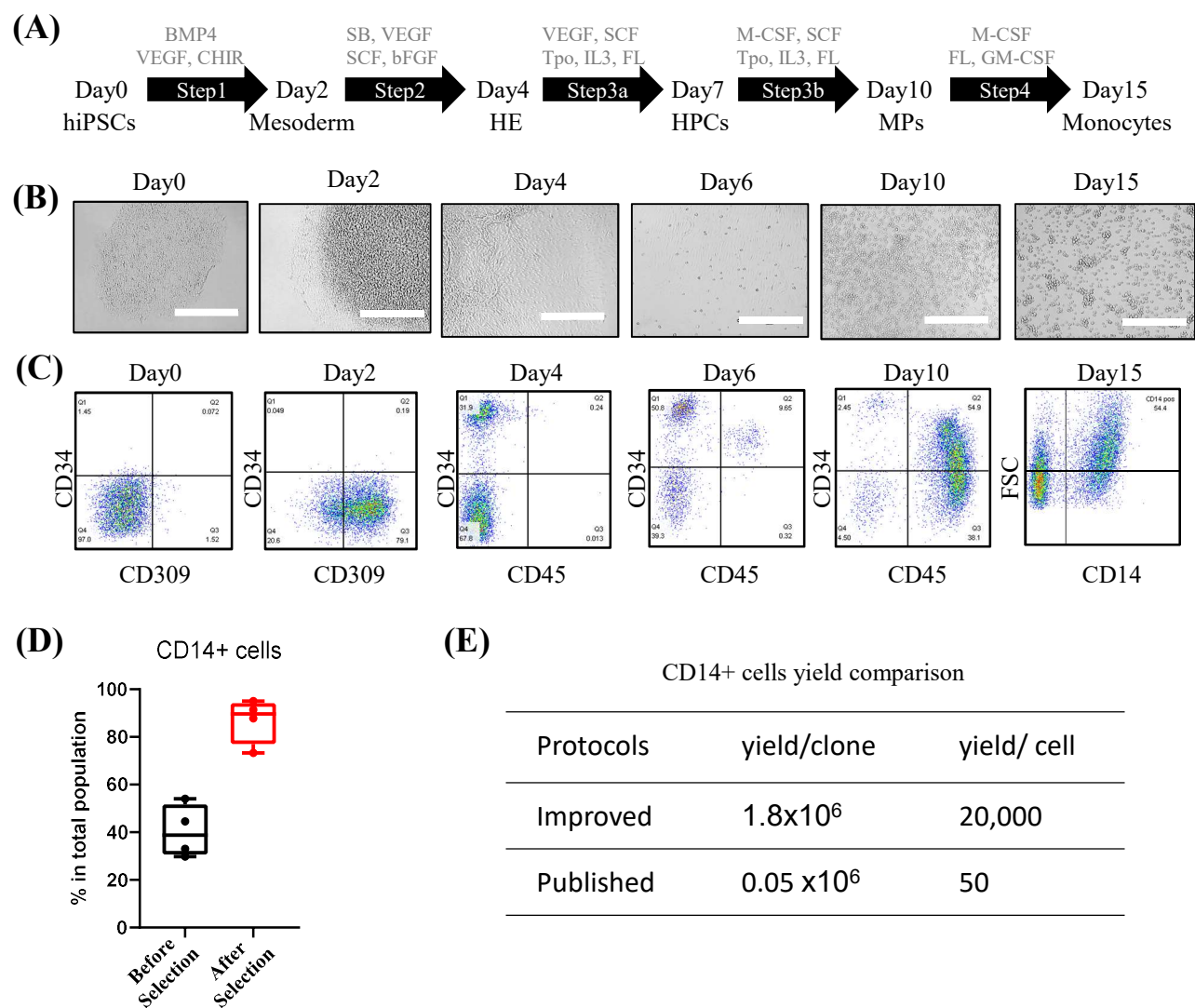

**Figure 1**

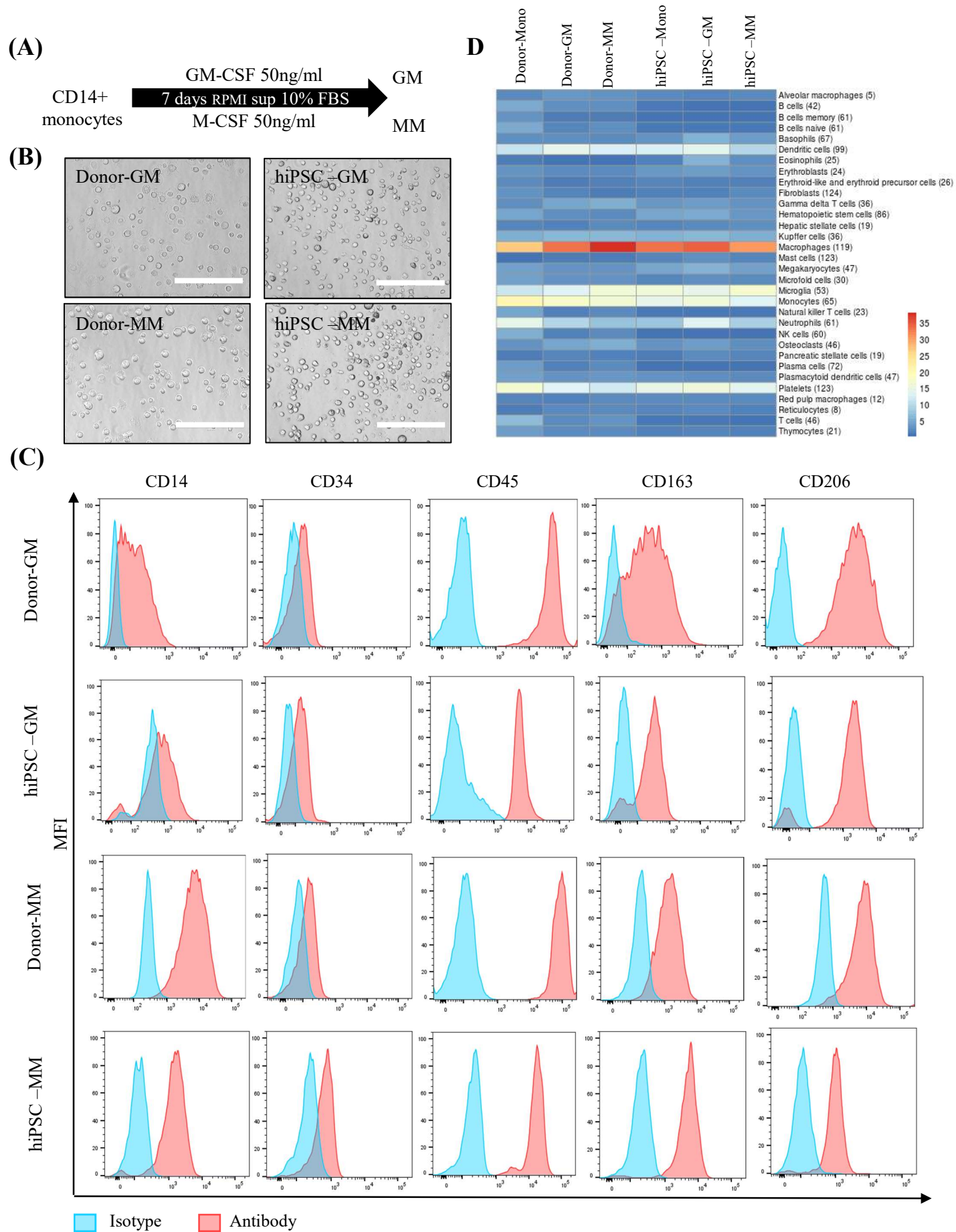

**Figure 2**

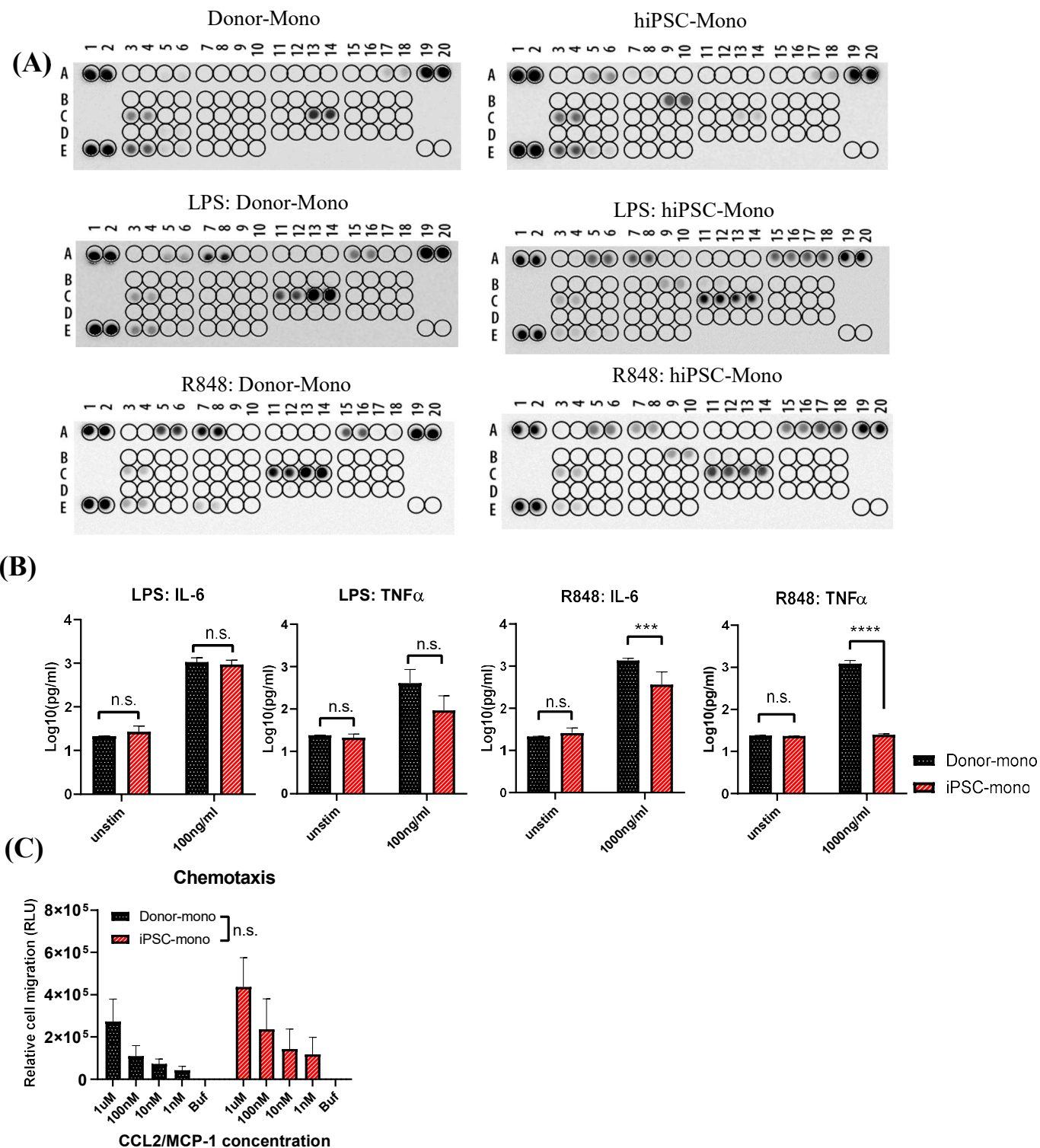

**Figure 3**

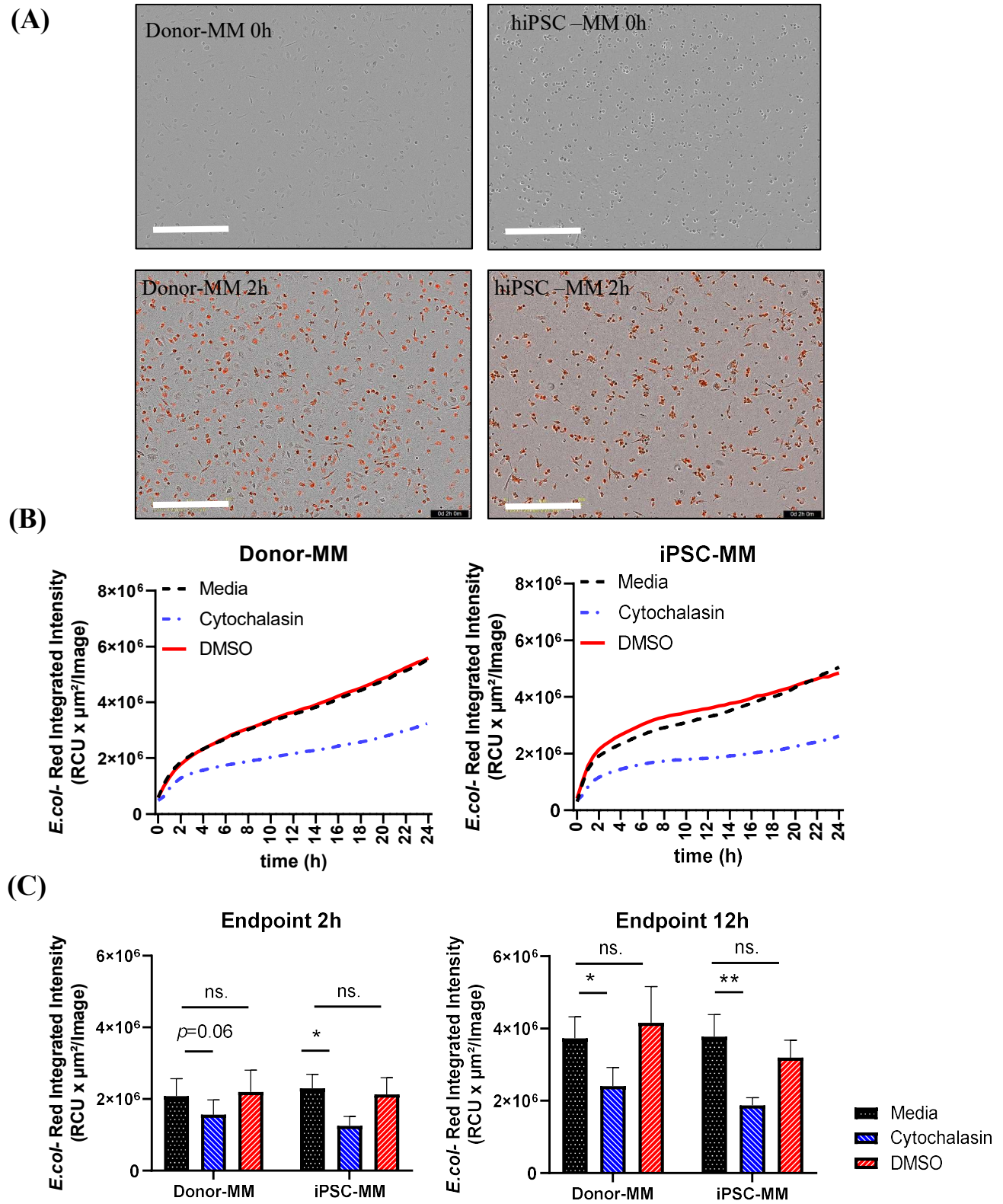

**Figure 4**

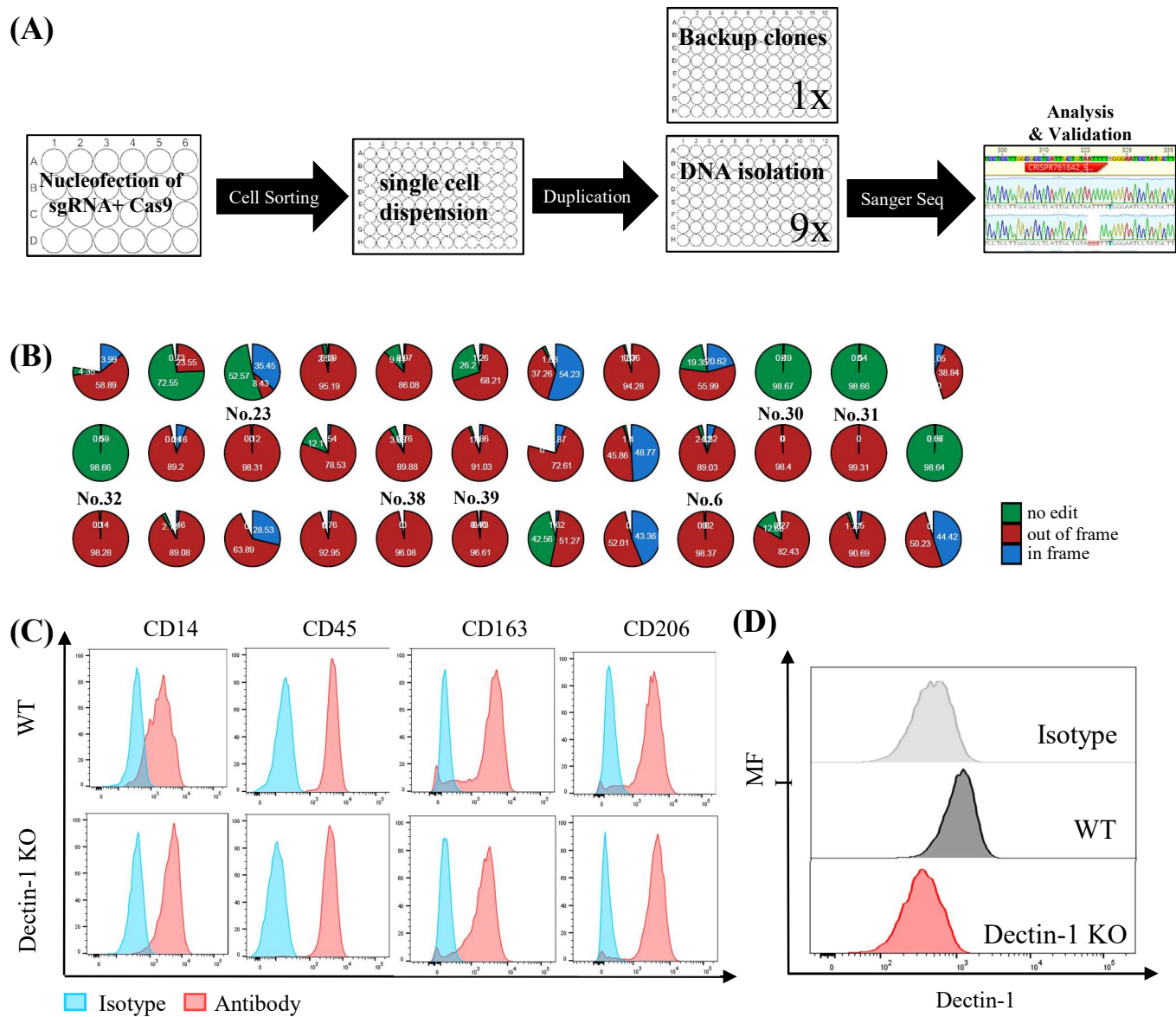

**Figure 5**

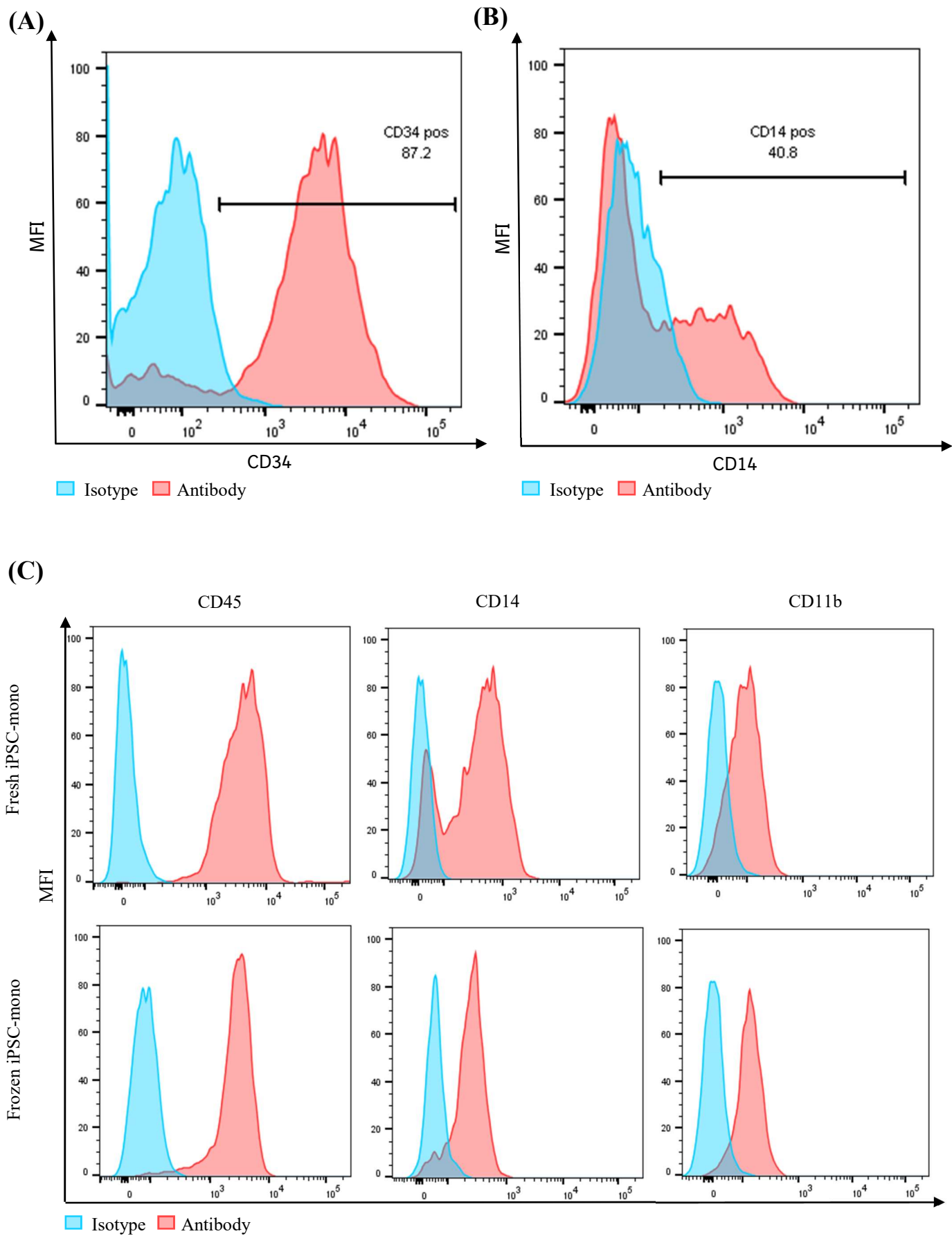

**Sfigure 1**

**(A)**

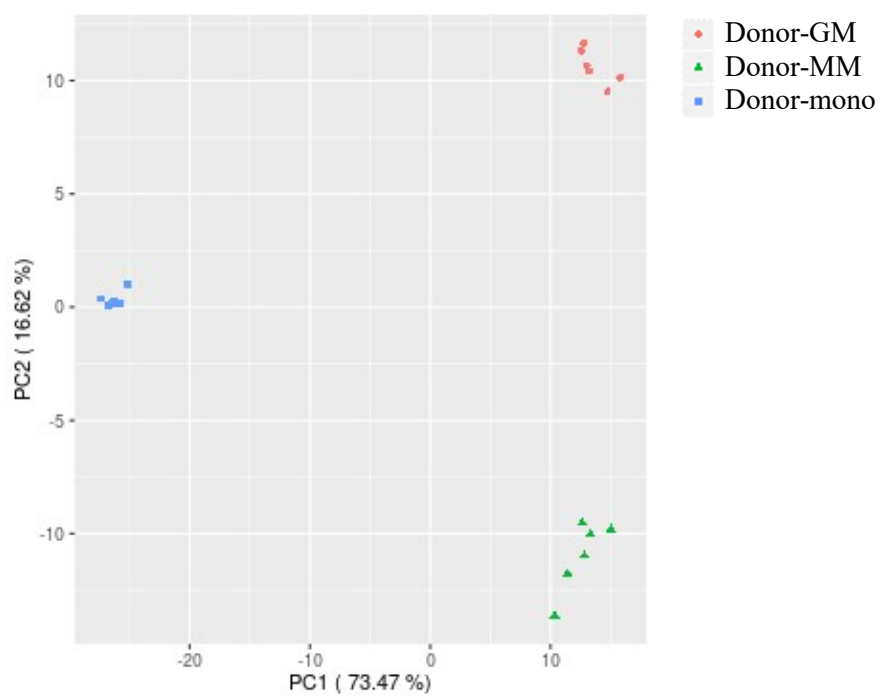

**(B)**

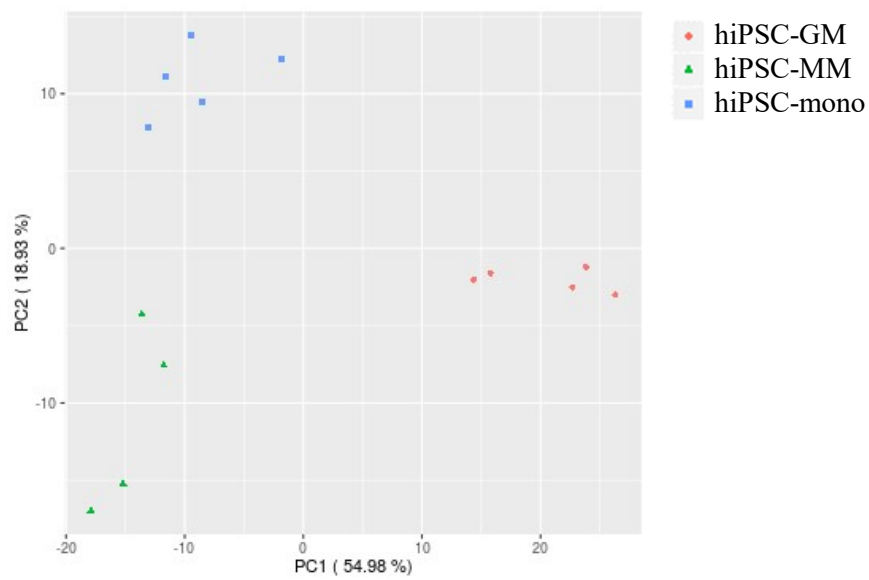

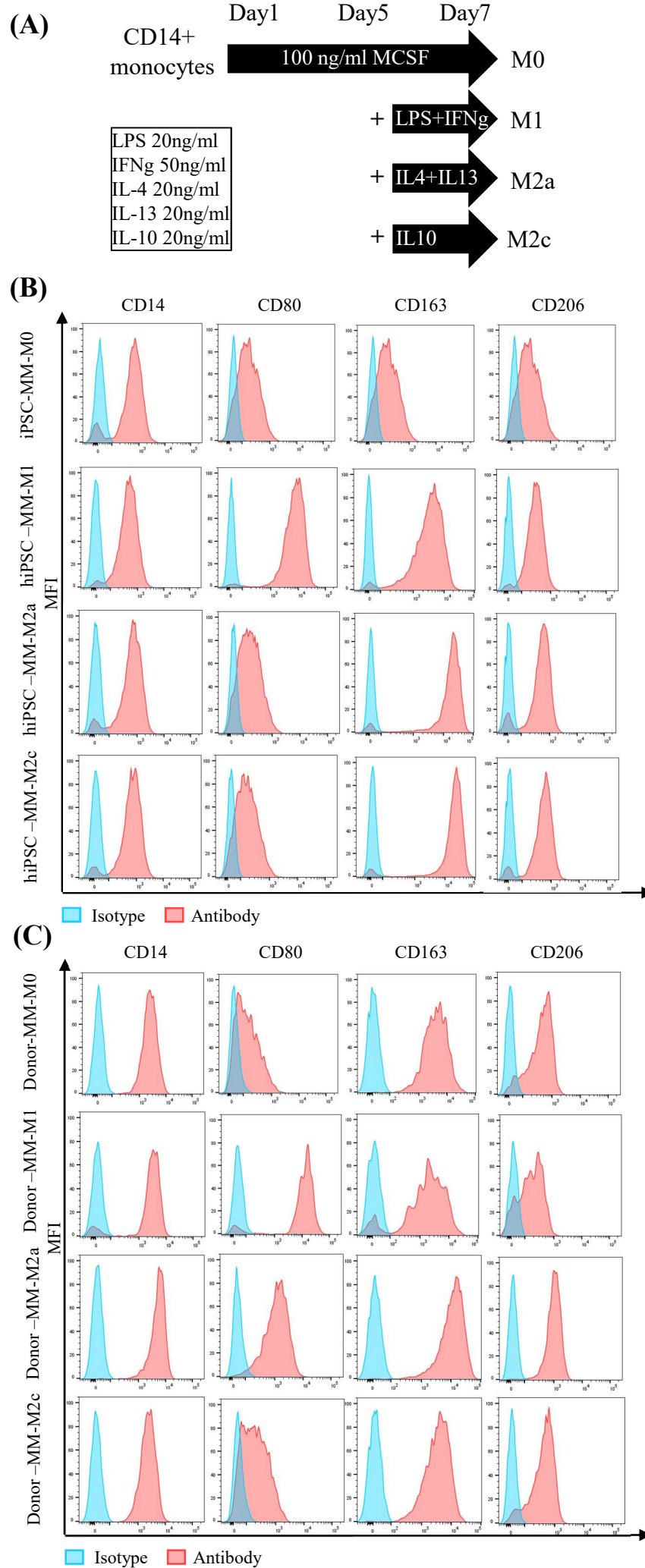

**SFigure 3**

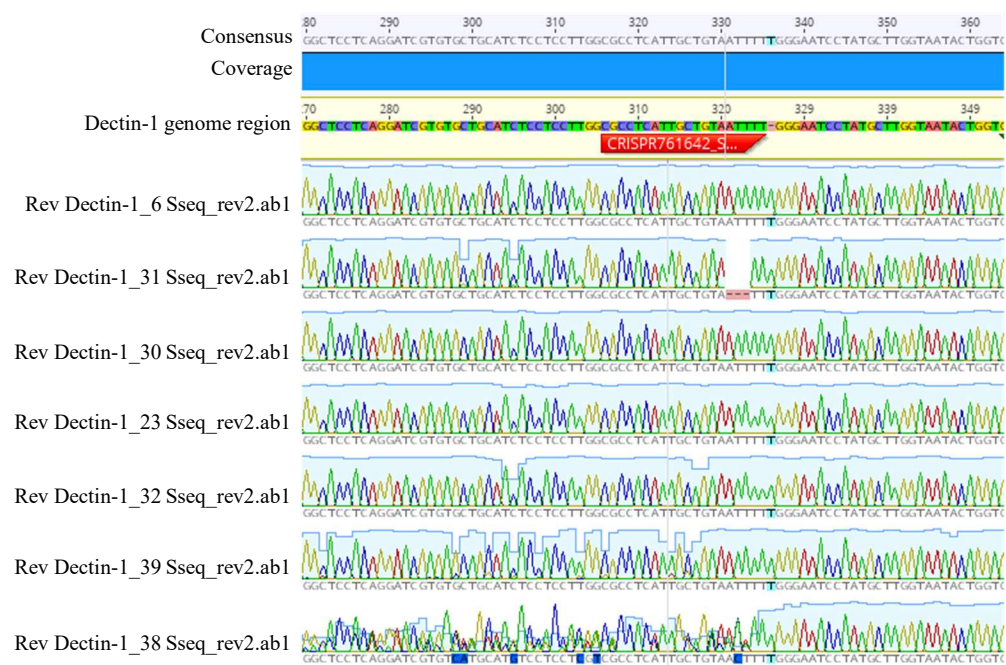

**SFigure 4**

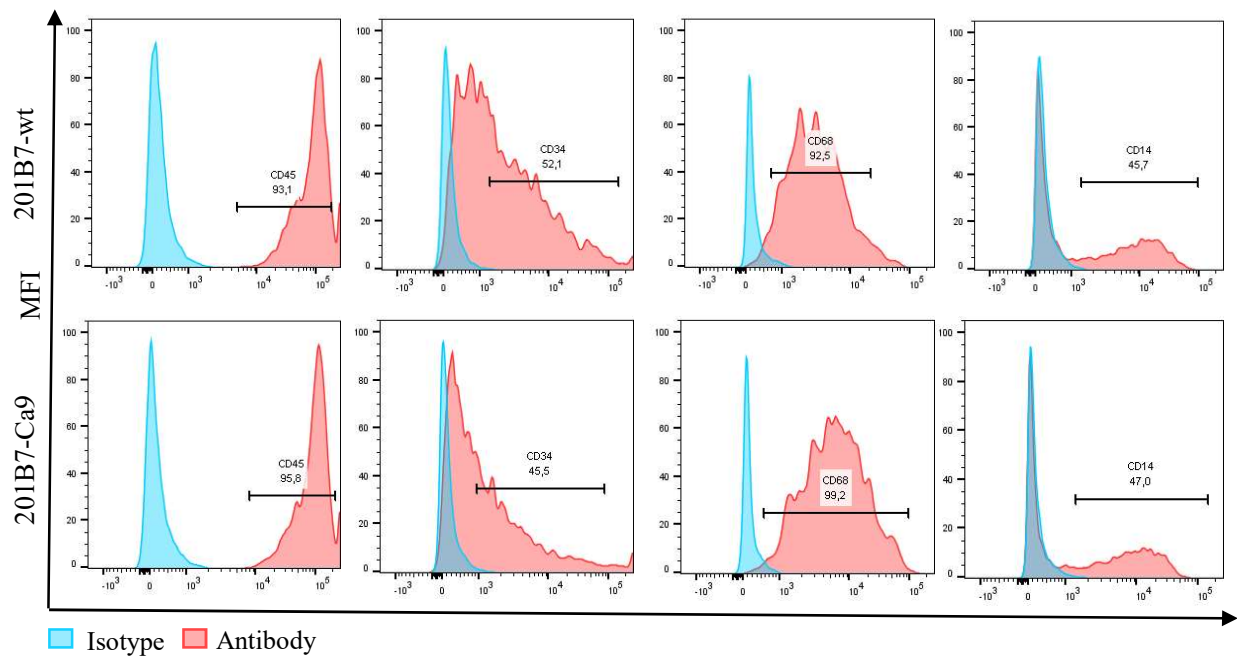

**SFigure 5**

Supplement: Supplementary file 6 [file Data_Sheet_1.PDF]
