# Supplementary material for: High-Yield Human Induced Pluripotent Stem Cell-Derived Monocytes and Macrophages Are Functionally Comparable With Primary Cells
Source: Front Cell Dev Biol. 2021 Apr 13;9:656867. doi: 10.3389/fcell.2021.656867 (PMC8080307; doi:10.3389/fcell.2021.656867)
Supplement: Supplementary file 7 [file Data_Sheet_2.docx]

Supplementary Material

# Supplementary Figures

**SFigure 1**. CD34 cell expansion and monocytes differentiation with frozen CD34 cells. (A) CD34 expression level and percentage after a week expansion. (B) CD14 expression of monocytes derived from frozen CD34+ cells. (C) CD45, CD14, CD11b expression level from fresh hiPSC-monos and cryopreserved hiPSC-monos.

**SFigure 2**. Principle component analysis shows a clear separation of monocytes, GM and MM macrophages in both Donor-(A) and hiPSC- derived cells (B).

**SFigure 3**. Comparison of hiPSC derived- and donor derived M0, M1, M2a and M2c. (A) Differentiation schematic overview of M0, M1, M2a and M2c. FACs characterization of markers for M0, M1, M2a and M2c macrophages from hiPSC derived (B) and donor derived cells (C) .

**SFigure 4**. Sanger Sequencing of the eight out-of-frame Dectin-1 KO clones at the Crispr/Cas genome editing region.

# Supplementary Tables

Table S1: the monocytes and macrophages all markers for cell type analysis

| Monocytes all markers | APOBEC3A, DYSF CMKLR1 MEFV FCGR3B PADI4 TNFRSF14, ADA2 MGMT GBP1 OAS1 PLSCR1 MX1 IL1RN IFIT1 IDO1 CXCL10 CLEC12A IFITM3 PSAP FCN1 RGS1 CD7 TET2 CD40 HCK GHSR ITGAX SELE TLR4 AR CXCR4 CD86 CCR2 CD14 CD33 ITGAM ACE FUT4 SELL CD163 FCGR1A, FCGR2B, ACP5 MRC1 IFIT3 S100A9, S100A8, S100A4, CLEC7A, CSF3R MNDA MS4A6A CD48 PRTN3 FCGR3A VCAN FN1 ADGRE1 CD44 CSF1R CX3CR1 ITGAL PECAM1 SPN |
| --- | --- |
| Macrophages all markers | CPM SLAMF9 PARP14 FGD2 RBPJ EGLN3 FGL2 ARL11 MMP12 FPR1 RAB20 FMNL1 CCL24 CD300A MYO1G DUSP5 SAMSN1 HILPDA SLC37A2, IFNAR2 HPGD CD5L FGR CYBB CD200R1 TYROBP SLC15A3, SYK ITGAL ITGAM CD14 FUT4 FCGR3A CD33 FCGR1A CD80 CD86 CD163 CCR5 TLR2 TLR4 ADGRE1 GPR34 TREM2 FABP4 S100A8 CHIT1 F13A1 CX3CR1 CXCL16 TGFBR1 SCIMP CD83 C3AR1 STAB1 MRC1 RAB7B SLCO2B1 NAAA JAML GPNMB CLEC4D ADAM8 LYVE1 PLTP VSIG4 MS4A4A MS4A6A CD180 GDF15 HFE CCR2 SNX20 GPR132 SLAMF7 NCEH1 C5AR1 CXCL2 CCL7 CCL2 IL1B IRF5 AHR GPR171 CCR7 DNASE1L3 CXCL1 NR4A3 CCL22 S100A4 MMP9 NRP2 CTSK CD36 LPCAT2 HPGDS SLC11A1 CLEC7A CYTH4 CD3E CD19 CD74 CSF1R LGALS3 CD68 UCP2 TREML4 CD200 GATA6 ITGAX PPARG RGS1 DAB2 P2RY6 MAF CLEC10A ADGRE5 CYP27A1 RUNX3 |
